# Off-Patent Biologicals and Biosimilars Tendering in Europe—A Proposal towards More Sustainable Practices

**DOI:** 10.3390/ph14060499

**Published:** 2021-05-24

**Authors:** Liese Barbier, Steven Simoens, Caroline Soontjens, Barbara Claus, Arnold G. Vulto, Isabelle Huys

**Affiliations:** 1 Department of Pharmaceutical and Pharmacological Sciences, KU Leuven, 3000 Leuven, Belgium; steven.simoens@kuleuven.be (S.S.); caroline.soontjens@student.kuleuven.be (C.S.); a.vulto@gmail.com (A.G.V.); isabelle.huys@kuleuven.be (I.H.); 2 Pharmacy Department, Faculty of Pharmaceutical Sciences, UZ Gent, 9000 Gent, Belgium; barbara.claus@uzgent.be; 3 Hospital Pharmacy, Erasmus University Medical Center, 3015 CN Rotterdam, The Netherlands

**Keywords:** tender, procurement, off-patent, biological, biosimilar, award criteria, switching, interchangeability, sustainability, competition

## Abstract

Background: In Europe, off-patent biologicals and biosimilars are largely procured by means of tender procedures. The organization and design of tenders may play a key role in the evolving biosimilar market, and currently, it is not fully elucidated how tenders for off-patent biologicals and biosimilars are designed and if approaches are aligned with sustaining market competition and societal savings for healthcare systems over the long term. This study aims to (i) explore the design and implementation of tender procedures for off-patent biologicals and biosimilars in Europe, (ii) identify learnings for sustainable tender approaches from purchasers and suppliers, and (iii) formulate recommendations in support of competitive and sustainable tender practices in the off-patent biologicals market. Methods: A mixed methods design was applied. A quantitative web-survey was conducted with hospital pharmacists and purchasers (N = 60, of which 47 completed the survey in full), and qualitative expert-interviews with purchasers and suppliers (N = 28) were carried out. Results: The web survey results showed that the organization and design of tenders for off-patent biologicals and biosimilars, and the experience of hospital pharmacists and purchasers with this, considerably varies on several elements across European countries. From the qualitative interviews, signals emerged across the board that some of the current tender approaches might negatively affect market dynamics for off-patent biologicals and biosimilars. The focus on generating short-term savings and existence of originator favouring tender practices were identified as elements that may limit timely competition from and market opportunity for biosimilar suppliers. The need to optimize tender processes, considering a more long-term strategic and sustainable view, was expressed. In addition, challenges appear to exist with differentiating between products beyond price, showing the need and opportunity to guide stakeholders with the (appropriate) inclusion of award criteria beyond price. Due to the variety in tender organization in Europe, a ‘one size fits all’ tendering framework is not possible. However, on an overarching level, it was argued that tender procedures must aim to (i) ensure market plurality and (ii) include award criteria beyond price (warranted that criteria are objectively and transparently defined, scored and competitively rewarded). Depending on the market (maturity), additional actions may be needed. Conclusions: Findings suggest the need to adjust tender procedures for off-patent biologicals and biosimilars, considering a more long-term strategic and market sustainable view. Five main avenues for optimization were identified: (i) safeguarding a transparent, equal opportunity setting for all suppliers with an appropriate use of award criteria; (ii) fostering a timely opening of tender procedures, ensuring on-set competition; (iii) ensuring and stimulating adherence to laws on public procurement; (iv) securing an efficient process, improving plannability and ensuring timely product supply and (v) safeguarding long-term sustainable competition by stimulating market plurality.

## 1. Introduction

Biological medicines represent a growing share of the total pharmaceutical spend, primarily driven by their high prices and increasing use and as such place a growing pressure on healthcare budgets [1]. In Europe, in 2018, over 30% of pharmaceutical expenditure was on biological medicines, totalling approximately EUR 53 billion [2].

With the expiration of patents and other exclusivity rights for numerous block-buster biological medicines, interest in the development and commercialization of biosimilars rose [3]. Biosimilars are products that are similar to an already authorized biological product, the originator product, with regards to quality, safety and efficacy [4]. The EU has the most mature biosimilar market at present, with 57 biosimilars approved for 16 distinct molecules across various therapeutic areas such as rheumatology, gastroenterology and oncology [5]. The biosimilar market is rapidly evolving and the number of approved biosimilars is expected to grow over the following years [6]. Biosimilars pose an opportunity for healthcare systems to foster competition following the originator’s loss of market exclusivity and lower spending on biological medicines while safeguarding safe and effective treatment. Of the total spend on biological medicines in the EU, 21% is now exposed to biosimilar competition (EUR 12 billion yearly) [2]. Biosimilar market entry has shown to lead to significant price reductions and increased patient access to biological therapies [3,7]. The 2020 IQVIA *The Impact of Biosimilar Competition in Europe* report showed that, based on list-price changes, biosimilar market entry has led to an overall 5% reduction in the total EU drug budget spending since 2014 [8].

On a pan-European level, moderate biosimilar uptake and considerable price reductions have been achieved. The experience of individual countries, regions and hospitals with biosimilars differs however considerably, which might be partly explained by the differences in biosimilar policies between and within countries [3,9].

To face budgetary pressures, cost-containment measures have been introduced by European payers to reduce pharmaceutical spending [10]. Tender practices are of specific interest as cost-containment measure in the context of off-patent biological and biosimilar procurement as they make use of supplier competition. Tendering is defined as a formal and predefined procedure in which multiple suppliers enter a contract competition, with the aim to select a best value for money medicine or medicines [10,11,12]. A tender procedure is generally applied to procure medicines when alternatives or equivalents for a specific medicine are available, which is the case for off-patent (originator) biological medicines and biosimilars. Hospital medicines, including most biologicals, should generally be procured by means of tenders in Europe. Public hospitals or non-public hospitals that are considered as bodies governed by public law should in principle organise tenders according to the harmonised EU rules on public procurement [13]. The EU rules are transposed into national legislation and apply to tenders whose monetary value exceeds a certain amount [14,15]. In tender procedures, price reductions beyond (mandatory) decreases at list-price level can be achieved. Together, these allow healthcare systems to optimize spending on off-patent biologicals and biosimilars. In addition to stimulating price competition, tenders may incentivize suppliers to compete on product or service differentiation, creating additional value for the patient and/or the care process.

When organized and applied in an appropriate way, tendering can be an efficient procurement mechanism, providing equal access to the different suppliers on the market and fostering competition between them, creating an opportunity for healthcare systems to contain expenditure and/or achieve savings that can be invested in other areas of care while possibly creating additional value for patients and care processes [12]. However, questions exist around the effective organization and application of tender procedures and significant variation exists in the organization of such tenders across European Member States, regions and purchasing groups [10,16,17,18,19]. The way how tender procedures are designed may have important implications on pharmaceutical market competition over the longer term [12,20,21,22]. Tender design elements such as the level on which tenders are organized, the number of winners, the tender duration and selection-and award criteria are important in this.

The importance of effective biosimilar competition for healthcare systems, together with emerging questions regarding the sustainability of tender approaches, the application of award criteria beyond price and the long-term viability of biosimilar commercialization [2,23], poses a timely opportunity to assess current tender practices for off-patent biologics and biosimilars and considerations regarding its possible influence on dynamics in the off-patent biologics market in Europe. 

This study aims to (i) explore the design and implementation of tender procedures by contracting authorities for off-patent biologicals and biosimilars and (ii) identify stakeholders’ learnings and components for sustainable tender approaches, to in the end (iii) formulate proposals in support of competitive and sustainable tendering practices, supporting long-term presence of different competitors and accompanying benefits for healthcare systems. Table 1 provides manuscript highlights.

## 2. Results

### 2.1. Survey Results—Organization and Design of Tenders for Off-Patent Biologicals and Biosimilars

In total, 60 hospital pharmacists and purchasers participated in the web-survey. The number of participants varied throughout the survey due to survey logic and participant drop-out. Forty-seven respondents completed the survey in full. Survey participants’ characteristics are shown in Table S1 in Supplementary Materials. In general, survey results showed that the implementation and design of tenders for off-patent biologicals varied on several elements.

#### 2.1.1. Perceptions about the Tender Organization

The majority of participants (61%) indicated their organisation to have moderate to extensive *experience* with tendering for biological medicines. Hospital pharmacists (88%), physicians (68%) and a procurement office (67%) were indicated to generally participate in formulating the tender conditions and subsequent product selection. A similar proportion of participants mentioned that differences (44%) and no differences (46%) exist between tender procedures applied for biologicals and small molecule medicines.

When tendering for biological medicines, 60% of participants identified *questions* about interchangeability and switching between biological reference products and biosimilars as challenging. Participants also identified the formulation of appropriate award criteria (25%), supply chain reliability (23%) and the formulation of criteria to select viable suppliers (19%) as challenges when tendering for biologicals. About one fifth of participants indicated to not identify specific challenges with tendering for biological medicines, different from those experienced with tendering for medicines in general. Full survey results are shown in Table S2 in Supplementary Materials.

#### 2.1.2. The Tender Design

The reported *average tender duration* varied substantially. Over one quarter of participants (27%) indicated that tender agreements are made for one up to two years. Approximately 20% of participants indicated that tender agreements last between six months and one year, and a similar number indicated tenders to last between two and three years. Tenders shorter than 6 months (12.5%) or longer, between 3 and 4 years (12.5%) appear less common. Approximately half of participants (55%) indicated that contracts can be *reopened after loss of exclusivity* of the tendered originator product. 

Almost half of participants (46%) indicated that tenders are generally awarded to a single *winner*. The same proportion of participants indicated that both single and multiple winner constructs are possible. Only 9% indicated to organize tenders with multiple winning suppliers.

Over half of participants (56%) indicated that the *physician’s voice* is incorporated in the tender procedure as being part of the tendering committee. According to 68% of participants, physicians can request a motivated exception to prescribe a different product than the tendered product. Only 10.5% indicated that physicians *maintain therapeutic freedom* to prescribe a different product than the tendered product. Full survey results are shown in Table S3 in Supplementary Materials.

#### 2.1.3. Application of Selection and Award Criteria

According to 68% of participants, no meaningful *differences* exist in the selection criteria applied in tender procedures for small molecule and biological medicines. Similarly, 60% of participants indicated that there are no differences in the award criteria for biological medicines and those for small molecule medicines while 33% made a distinction.

In terms of applied *selection criteria* (when applicable), 27% indicated to consider the financial viability of the supplier. One fifth of participants indicated to consider the supplier’s reputation and the supplier’s production capacity. To a lesser extent, participants indicated to consider the supplier’s track record of previous tenders (16%), previous collaboration (12%), the duration that the supplier already markets the product (8%), the market share of the product (6%) and the supplier’s investment in academic research (4%).

In terms of applied *award criteria besides price*, the product’s registered indications (49%), the product’s stability/shelf life (45%), the product’s delivery device (35%) and the packaging (35%) were indicated to be generally considered. In terms of award criteria related to supply, 41% of participants indicated to consider the supply conditions and 29% the emergency delivery and 24/7 reachability of the supplier. Almost a quarter (22%) of participants indicated to award on additional efficacy and/or safety data (in addition to the data required for regulatory approval, such as clinical data in an additional patient population, or switching data). Value added services (e.g., supporting educational activities, product training programs, information brochures for HCPs or patients about the product, support with switching from the medicinal product previously used) (18%), customer support (14%) and expenses incurred from switching from the previous winner (6%) were considered to a lesser extent.

The *relative weight given to price* when awarding the tender varied among participants. The majority of participants indicated that a certain weight was given to award criteria besides price (predominately awarded on price (38%), a 50/50 distribution between price and other criteria (19%), predominately on other criteria besides price (19%)). Approximately 20% of participants indicated tenders to be awarded entirely on price.

When formulating *award criteria*, a large number of participants indicated to do so in collaboration with or advice from experts within their own organization (70%). Over half of participants indicated to base themselves on previous experience and almost half to base themselves on national or European guidelines. Thirteen percent of participants indicated to formulate award criteria in collaboration with or advice from (one of) the suppliers. Full survey results are shown in Table S4 in Supplementary Materials.

#### 2.1.4. Interchangeability and Switching Considerations in the Context of Tenders 

For the formulation of the tender, over half of participants deemed biosimilars interchangeable with their reference product, while 28% believed this depends on the product class and 13% indicated that biosimilars and their reference product are not interchangeable. The majority of participants (68%) indicated that biosimilars and the reference product are grouped in the same lot. According to 43% of participants, no *difference* is made between bio-naïve patients and patients already under treatment with the biological medicine when tendering for biological medicines, with 36% indicating that a difference is made. When the patient already undergoes treatment with the previous winner, approximately half of participants indicated that the option is foreseen to keep patients on therapy with the previous winner. This was indicated to be realized via a multiple winner tender, i.e., there are multiple winners, and one of them is the previous winner (29%), direct procurement of the previous winner (42%) or via an existing contract with the previous winner (21%). Full survey results are shown in Table S5 in Supplementary Materials.

### 2.2. Interview Results—Considerations Regarding the Design and Organisation of Tender Procedures

In total, 28 expert-interviews were conducted. Tables S6 and S7 in Supplementary Materials provide an overview of interview participants’ characteristics.

#### 2.2.1. Considerations Regarding Tender Design Elements

##### Dividing Product Volume among Suppliers—Ensuring Market Plurality

Presently, tenders are often organised on a single-winner basis, in which the total tendered volume is awarded to one supplier. A *single-winner tender design* generally leads to significant discounts, certainly if the product volume is significant such as in national single-winner tenders. The generated initial price pressure has proven advantageous for healthcare systems to realize immediate large savings. However, awarding total market volume to a single winner excludes non-winning competitors from the market for the duration of the tender contract. While price-driven, single winner tenders generally translate in welcomed large initial savings for healthcare systems, these might decrease supplier plurality in the market. A proliferation and continuation of the single winner-takes-all approach may as such lead to reduced levels of competition. In addition, relying on a single or limited number of suppliers may impact the continuity of patientcare in case of product shortages. Large volume, single-winner tenders may in addition imply a potentially large time and product write-off for contenders who did not win. 

Dividing the market among multiple suppliers, providing a commercial opportunity for several suppliers and ensuring *plurality in the market*, was the single most recommended intervention by interviewees towards creating more sustainable tender practices. Some hesitations were expressed by purchasers, as the organization of multiple winner tenders increases the complexity of tenders and product management in the hospital. Healthcare systems and purchaser authorities need to be equipped to accommodate and effectively organize such a multi-winner tender structure. Besides this remark, both purchasers and suppliers broadly voiced their support. Awarding tenders to multiple winners may also contribute to lower price pressure due to the smaller product volumes. In addition, it provides price reductions on all tendered products and may possibly increase the physician’s therapeutic freedom to choose between different products, as such avoiding physicians using a higher-priced non-tendered product. The availability of multiple commercial products on the market may further help to mitigate supply chain issues.

*Various scenarios* could be explored and applied to ensure market plurality, depending on the market size and product volume. Multi-winner tenders (i.e., the tender is awarded to multiple bidders) can be organized or markets can be divided into multiple commercial single-winner opportunities (e.g., on hospital network or regional level).

To effectively organize a multi-winner tender, interviewees argued that some *conditions* need to be fulfilled. First, the tendered volume on purchaser level needs be large enough to be divided among multiple bidders. Second, from the perspective of the purchaser, purchasing capacity would need to be consolidated to increase the feasibility of organizing multi-winner tenders, as this may add to complexity and workload. Third, suppliers should be provided with a guarantee regarding the allocation of volume per supplier. A clear volume estimation per winner is needed to allow them to manage their supply chain and formulate a competitive bid. Multi-winner scenarios in which the first winner is the utilized product and other winners serve as back-up in case supply issues would occur with the first-ranked winner are to be avoided. 

In countries where tendering takes place on hospital (group) level (typified by small volumes and generally small procurement teams), single-winner tender structures may be a more efficient route while still stimulating competition as multiple opportunities to win volume exist across the market. 

Dividing the market volume among multiple winners on a central or regional purchasing level should not necessarily translate in the availability of multiple products on an individual hospital level. The different winners may be allocated to certain regions or hospitals, which is for example the case in England. 

The advantages and conditions related to the organization of multi-winner tenders are outlined in Table 2.

##### Tender Award Criteria—Ensuring a Fair Design and Application

Purchasers are encouraged to award a tender based on the Economically Most Advantageous Tender (MEAT) principle, including qualitative elements linked to the tender-subject beyond price, as outlined in the EU Procurement Directive 2014/24/EU [13,24,25]. Tender procedures that solely or mainly focus on price, while delivering savings in the short term, may lead to price erosion and lower the number of competitors over the longer term. Interviewees cautioned that this could ultimately result in de novo market consolidation and increased prices in a given market.

Lowest bid procedures should be avoided, and suppliers should aim to compete sustainably *on additional elements*. Multiple European trade organizations (both originator and biosimilar oriented associations) and also the European Association of Hospital Pharmacists (EAHP) underwrite the practice to include criteria beyond price in tender procedures [20,21,26,27,28], as such awarding the best-value biological(s). An overview of position statements of these organizations is made available in Table S8 in Supplementary Materials. The inclusion of award criteria beyond price can lead to benefits for the patient (e.g., less painful injection) or the broader organization of care (e.g., facilitating efficient handling by means of ready-to-use preparation or pre-filled syringes). Including additional criteria besides price can furthermore contribute to countering steep price erosion identified in price-only tenders, as this would stimulate to suppliers to innovate and sustainably compete on value-adding criteria.

Four main *challenges* related to including additional award criteria emerged from the interviews. First, the inclusion of award criteria besides price appears not to be routinely included in tenders for off-patent biologicals and biosimilars. According to interviewees, price remains often the sole or dominant differentiator in tender decisions.

Second, stakeholders appear to have questions on how to exactly formulate and apply these criteria. Both purchasers and suppliers mentioned difficulties with translating the MEAT principle to applicable award criteria for off-patent biologicals and biosimilars. As stipulated in the EU Public Procurement Directive, criteria should be compliant with the principles of transparency, non-discrimination and equal treatment to allow an objective comparative assessment [13]. Any criteria that could be perceived as anti-competitive or introduce bias should be excluded from inclusion. Further, only criteria that are related and proportionate to the subject matter of the tender should be included. Caution should be exerted regarding requesting or offering additional services or benefits. In case these are not directly related to the subject matter, these should be strictly avoided. Some interviewees mentioned for example the offering or requesting of research funding. This leads to the third identified challenge related to the application of award criteria.

It appears that in some cases where additional criteria are included; these may *a priori* favour the reference product or disadvantage the biosimilar. For example, including an award criterion on the length of product market presence would structurally disadvantage recently launched products, i.e., biosimilar alternatives, compared to the reference product. Including such an award criterion could therefore be considered as an unreasonable expulsion of competition. Moreover, criteria that are not directly related to the subject-matter can steer the decision-making on non-product related factors and especially when these are disproportionally weighted in the decision. Additional product-related services are mentioned to be interpreted broadly in some instances. Requesting or offering bonuses or benefits beyond the scope of the product, such as research grants and conference support, should be strictly excluded. In Table 3, an overview of the types of criteria that should be avoided is shown. In Belgium, it was mentioned by stakeholders that the possibility to provide free goods via medical need programs might also disadvantage biosimilars, as these cannot be applied for if already been granted for the reference product.

Fourth, suppliers expressed difficulty in terms of determining award criteria that would allow to truly differentiate and compete on. It was mentioned that the applied award criteria beyond price often can be relatively easily fulfilled by all suppliers. In such case, including additional criteria increases the effort and cost for the supplier, without playing a differentiating role in the allotment. Interviewees also mentioned that most criteria only temporarily offer a certain differentiation. With the increasing experience with biosimilars, the need for services in terms of educational switch support may for example wane. Moreover, competitors will prepare to meet differentiating additional award criteria in the subsequent tender rounds. Due to the comparable nature of reference biologicals and biosimilars, it may prove challenging to develop criteria on a product level that could offer differentiation over a longer term. Purchasers also alluded to the fact that the inclusion of additional award criteria should serve to drive actual added value rather than complicating interchangeability of products. To allow for appropriate evaluation of possible differentiating elements such as injection pain of the product, appropriate supporting data are needed.

From both the supplier and purchaser perspective, there is a strong request for a *framework* with general principles regarding the structuring and application of award criteria. In order to stimulate the inclusion of criteria besides price and ensure a correct application, guidance should be drafted to support involved stakeholders, especially purchasers with formulating their tenders. The EU Public Procurement Directive has set out a frame in which Member States and purchasers can operate. Further action may be needed to ensure proper translation and application of MEAT in practice on Member State and purchaser level. Relevant experts should be integrated to identify appropriate award criteria. In countries where procurement is organized on a local or individual hospital level, it may be useful for governments to provide such guidance to purchasers. Here, a flexible or semi-structured tender template could be designed to guide purchasers. Room for flexibility should be foreseen, to allow tailoring based on product-specific considerations and strategic differentiation. Such an award criteria template could be piloted with collaboration from tender authorities and governments.

Only additional criteria that drive *meaningful product differentiation*, leading to an advantage for the organization of care and/or the patient should be included. Criteria could include considerations related to various elements such as supply, packaging, product presentation, storage, reconstitution and easiness of use, licensing and product-related services. To give an example, several purchasers deemed data from stability studies a possible important differentiator for products that require reconstitution. An overview of criteria that can be taken into account is shown in Table 3. Award criteria besides price should also be *proportionally rewarded* based on the additional value created. This should enable criteria besides price to truly play a role in the allotment. Suppliers mentioned that actions are needed to include these additional criteria in the tender, otherwise potential differentiation strategies could be done in vain from the supplier perspective.

Finally, award criteria need to be *transparently formulated*, and it must be clear to participants how these will be evaluated, i.e., which weight will be given to the criteria in the decision-making, and how will they be scored.

Arguments were made that a shift to the inclusion of additional decision-making criteria may gain more attention in future tenders. As first tenders focussed on steep discounts, further discounting opportunities are finite. Including other award criteria may increasingly help differentiate between products.

##### Tender Frequency and (Re-)Opening of Contracts—Ensuring Timely Competition

The time between the first possible use of a biosimilar after loss of exclusivity of the corresponding originator product and its actual use should be minimized. In addition to streamlining pricing and reimbursement procedures, a *timely opening* of tender procedures is essential to avoid delays in competition and ensure swift market opportunity for biosimilar alternatives. In addition to ensuring commercial opportunity, a timely opening of tenders should be stimulated to generate savings for healthcare systems as soon as possible.

Several interviewees mentioned that in some cases tenders are opened with a *significant and unnecessary delay*. Contracts with the supplier of the reference product that still apply at the time of biosimilar market entry could possibly explain a delayed tender opening. It was hypothesized that in some cases these contracts were strategically agreed prior to biosimilar market entry to as such extend the originator’s market exclusivity artificially. Another possible explanation, which was also mentioned by purchasers, links to the fact that an overview of upcoming loss of exclusivities of reference products and biosimilar market entry dates on governmental and/or purchasing level lacks. 

To ensure timely competition, healthcare systems and purchasers should *anticipate and prepare* for biosimilar market entry well in advance. Horizon scanning should be performed to identify the upcoming loss of exclusivity of reference products and anticipated biosimilar market entry dates. In addition to early preparation for the opening of tenders upon loss of exclusivity of the reference product, purchasers should coordinate contracts with the originator prior to its loss of exclusivity, taking the future entry of biosimilars into account. The length of the contract with the originator prior to biosimilar market entry should thus be set accordingly and preferably/compulsory include a clause that allows reopening if a biosimilar alternative enters the market, to avoid such blocking contracts at the time of biosimilar market entry.

In essence, competition should be realized as soon as possible, providing commercial opportunity, onset savings and possibly additional benefits. Below, different *approaches* are suggested that could be suitable to translate the timely opening of tenders into practice. First, healthcare systems and purchasers could set a certain term in which for existing public contracts a new tender procedure would need to be organised. This term could be included in legislation and made mandatory, such as is the case in Italy [31]. Here, regional authorities are obliged to re-open supply agreements within 60 days after entrance of the biosimilar medicine to the market [31]. A few interviewees mentioned that it should be made (more) clear if reopening is expected with every new entrant. Opening a tender upon market entry of the first biosimilar(s) could challenge market opportunity for subsequent biosimilar entrants for the same product. On the other hand, launching a new tender upon market entry of each subsequent biosimilar should be avoided as a reopening would increase workload, possibly involve repeated switching and increase uncertainty related to the product volume. The latter may prove especially challenging for suppliers. Installing a shorter-term tender (e.g., 6 months) immediately upon market entry of the first biosimilar competitor(s), combined with a longer subsequent tender duration agreement (12–24 months) once the market has further matured in the number of competitors, could be an appropriate alternative when multiple biosimilars are expected to arrive to market in a staggered way. The combination of an on-set short term tender with a subsequent longer one, would allow direct competition, leading to immediate savings for the payer and commercial opportunity for the first biosimilar supplier(s), while avoiding a closed market for subsequent suppliers for a considerable length of time. Once the market has further crystalized in terms of number of available products (e.g., in 6 months or a year depending on estimated market entry dates), tenders for existing public contracts could be reopened. Such a combined approach is for example applied by the central purchasing body Amgros in Denmark.

The appropriate approach in terms of tender timing and frequency could be determined based on the *market-specific circumstances of the product*, such as the expected number of competitors and their anticipated dates of market entry. Tenders that are organized on a quarterly basis might create a high administrative burden for both purchasers and suppliers in addition to being undesirable from the switch perspective. On the other hand, tenders with a duration beyond two years may restrict competition from other suppliers over the longer term. Generally, a tender duration between 12 and 24 months is considered desirable in terms of stimulating market dynamics, while considering feasibility and avoiding a regular switch of patients by interviewees.

In countries where tendering is organized on a regional, purchasing group or hospital level, tender procedures could open up at varying times throughout the year, to spread commercial opportunity for suppliers and accommodate manufacturing capacity. Such a *rolling system* is in place in England, with the Tranche frameworks opening every six months in another one of the four regions [32]. A *specialist procurement office* can play an important role in organizing and coordinating the timing and duration of tender procedures for products with biosimilar competition.

A *financial stimulus* (positive or negative) could also be considered to motivate purchasing bodies/hospitals to timely organize tenders, aligning the incentives of the purchaser with these of the overall healthcare system (savings for healthcare budgets, and/or premium payers/patients). For example, in Belgium the reimbursement agency lowered the reimbursement for biologicals for which a biosimilar alternative exists with 15% to hospitals [33,34]. As margins on the negotiated price difference between the tendered price and reimbursement limit can be retained by hospitals, hospitals are motivated to organize competitive tenders to procure medicines at low net prices [35]. A similar construct exists in the Netherlands, where health insurers reimburse hospitals the list price of biologics with biosimilar competition only in part, anticipating savings based on discounts that hospitals negotiate in tender procedures [36].

##### Supply Conditions—Increasing Volume and Predictability to Ensure Continuity of Supply

Tender procedures need to be *efficiently managed*, to increase predictability and plannability for the supplier, which can in turn guarantee timely product supply for the purchaser. Special attention needs to be paid to the setting of product volume, lead time and supply agreements.

First, *increasing predictability* regarding the tendered volume is of benefit for both the purchaser and the supplier. It provides suppliers the ability to accurately assess the economies of scale in their bid, increase the ability of suppliers to participate in tender bidding and manage production. The latter may help to avoid undue pressures on the supply chain.

This includes *setting of reliable estimates of the volume* to be supplied, with guaranteeing a minimum volume and defining a maximum cap. Moreover, in the context of multi-winner tender structures, a clear and guaranteed (division of) volume was considered a prerequisite to allow participating bidders to plan accordingly. In addition, *clinical use guidelines* should be reviewed and revised, if needed in this context, following introduction of biosimilars to allow purchasers to correctly estimate (potentially increased) volumes for tenders. Covering an unexpected increase in demand may be difficult, as it is complex and lengthy to increase the production scale due to the complex manufacturing process of biologicals. In case no minimum volumes would be guaranteed, tenders could lead to a risk of unused stock and issues with scaling [37]. Suppliers with overstock may go for highly competitive offers in pending or subsequent tender procedures, which may lead to unsustainable market dynamics.

Second, the time between the announcement of the winner(s) and the start of the contract (first delivery), also called *lead time*, is in some instances (deemed too) short, making the first supply deadline challenging. Lead times between minimum three to six months should be respected to support the supplier’s supply chain management (taking into account that decisions regarding for example packaging cannot be easily re-allocated to other markets), as such reducing the risk of delayed deliveries and shortages. In general, *early communication* regarding the timing of tender procedures and expected volumes should be promoted. 

Third, although fortunately no interviewees reported supply inabilities having occurred (yet) in the context of off-patent biologicals and biosimilars, the *hedging agreements for possible supply problems* are a point of consideration. By contract, suppliers are generally obliged to compensate the difference between the tendered price and the price at which the alternative product is offered by a competing supplier, often the list price, to remediate the supply issue. Although the burden of securing and financing an alternative product should naturally not be placed on the purchaser, the supply conditions should be set in such a way that they are manageable for suppliers to achieve, i.e., based on early and accurate communication regarding timing and volume of tender. Moreover, penalties should be proportionate to the contract value and the cause of the inability to supply (force majeure/external reasons for which could not be controlled), ensuring a fair balance of risk and reward for the supplier. For example, in France, penalties are based on list price and not net price, which might lead to an unbalanced risk and reward [37]. Suppliers might decide not to participate in tenders where penalties are disproportionate, leading to reduced competition. *Dialogue* between purchasers and industry should be stimulated to establish manageable supply conditions and balanced penalties. 

In the case of a supply issue in a single winner tender market, other manufacturers might not be able to cover the sudden demand and remedy a potential shortage as their production may be reduced or discontinued [37]. *Multi-winner tenders* might thus also be preferred in the context of mitigating the risk of possible medicine shortages, increasing the opportunity to source the product with another supplier. Purchasing strategies that result in steep and perhaps over the longer term unsustainable price reductions may also impact supply, as companies might economize on services such as the presence of strategic stocks. It was argued that focussing on price only may impact additional services and as such the quality of the supply chain.

A *joint tendering initiative* was set up between Norway, Iceland and Denmark in 2019 in response to the growing challenges with regard to supply security, especially for older medicines [38]. Such contracts with large volumes are likely to be prioritized by pharmaceutical companies because of the potential large gain. Such evolution may however be less advantageous on a broader level as it further consolidates the market. Cross border procurement should be reserved to situations where purchasing and supply of products can alternatively not be ensured.

#### 2.2.2. Considerations Regarding the Organization of Tenders

##### Considerations Regarding Transparency about the Tender Procedure and Price

*Transparency* in tenders should be stimulated *throughout the procedure*. Prior to the start of the procedure, at the time of publishing, the tender format, including the eligibility and award criteria and the relative weight that is awarded to these, should be clearly communicated. Upon awarding the contract, feedback should be foreseen to the participating supplier regarding the allocation decision and their scoring. Moreover, the obligation to publish the contract award notice for contracts for which prior announcement is not needed, e.g., for exclusivity contracts (negotiated procedure without prior call for competition) with the incumbent/patent holder prior to biosimilar market entry, should be complied to increase transparency towards the biosimilar entrants. Managed entry agreements (MEAs) ask for specific attention in this regard. The confidential and opaque nature of MEAs, with also the concealment of the patent expiration date of the reference biological, hampers the market entry of biosimilar alternatives. Confidentiality provisions should be addressed to improve the design and transparency of such agreements [39]. 

A few interviewees argued that a *Best and Final Offer (BAFO) procedure*, which involves a negotiation or clarification on a first written offer, after which bidders are invited to submit a final offer, or any route that would provide a certain supplier to submit a second (informal) bid to surpass the offer of competitors, should be avoided. This practice may provide leeway for suppliers and purchasers to include offers or request elements that are outside the scope of the tender subject matter, as transparency lacks during the final offer made, and impact the equal opportunity setting.

The size of rebates in tender procedures is noted to vary considerably (depending on market maturity and tender volume), ranging between 10% and 90% of the list price [8]. In terms of *price transparency*, actual contract prices are seldom publicly available, hampering the insight in the size of actual rebates [8]. In Norway, where prices were made public from 1995 until 2017, prices from tender procedures are no longer made public [40]. Industry might be willing to provide larger discounts when tender prices remain confidential and list prices un-impacted. *Providing confidential discounts in tenders* is likely to be preferred over pricing strategies that lower the medicine’s list price. List prices are often included in external reference pricing systems, acting as benchmark in terms of list price regulation in other European countries. Confidential tender discounts avoid such leverage in price negotiations in other jurisdictions.

##### Switching Considerations in the Context of Tenders—Clinical Data, Cost, Physician Freedom and Guidance

Increasingly, guidance statements from EU Member States support that prescribers can safely switch patients from a reference biological to its biosimilar [41]. Requesting *additional switching studies* could create an extra barrier for biosimilar developers and may advantage the incumbent, who does not need to gather such data, in tender procedures.

Similarly, determining and including *a switch fee per patient* in tender procedures would disadvantage the biosimilar competitor, as an additional price lowering of for example 5% would be needed to offset the switch fee. Most purchasers argue that the *cost of switching* is marginal compared to the savings that are generally generated in a tender and will as such not play a decisive role in tender decisions. Originator companies may have however some leverage in the broader procurement context, as the price of the originator product that may needed to be purchased to treat the rest population (patients that remain under treatment with the reference product) can be raised by the company in case they lose the tender contract. This could limit or offset the discount realized in the tender procedure, where the originator competes with its biosimilar (alternatives).

In addition to *guidance* regarding interchangeability and switching by authorities [41], purchasers and hospitals should receive *practical support* regarding the use of biosimilars and switching in clinical practice. Practical barriers associated with biosimilar use and uncertainty among stakeholders should be lowered. For example, in England, the NHS set-up different initiatives to educate stakeholders about biosimilars and provide guidance, with the aim of supporting safe, effective and consistent use of biologicals, including biosimilars [42,43]. In the Netherlands, some health insurers have applied a differential reimbursement, reimbursing hospitals at a premium for using biosimilars, as a benefit share between insurers and hospitals, with the aim of compensating hospitals for the time and cost investment associated with a switch [36].

##### Collaboration and Communication in the Context of Tenders

Collaboration among stakeholders could be stimulated to ensure the development of more sustainable tender practices. First, early involvement and agreement between the internal stakeholders at the purchasing side (i.e., dialogue between purchasers, hospital pharmacists, physicians, nurses, etc.) regarding the modalities of the tender is believed to be essential. In hospitals, this is generally organized in a Drug and Therapeutics Committee. In countries with a centrally organized procurement such as Denmark and Norway, procurement bodies work together with specialist groups or expert committees. This approach is argued to result in good agreement of physicians to prescribe the tendered medicine.

Second, collaboration *among purchaser(s) (groups)* can increase negotiating strength and add to the consolidation of expertise, professionalism and capacity which is needed to conduct efficient and high-quality tenders. Third, *communication between industry and purchasers* should be stimulated. Increased dialogue could reduce supplier uncertainties and increase efficiency for the different stakeholders involved, establishing a balanced shared risk and reward between suppliers and purchasers. This could be pursued both on the supplier and purchaser level, in the context of specific procedures (preliminary market consultations, with the prerequisite that every supplier is treated equally and receives the same information) and by stimulating dialogue between umbrella industry and purchaser associations. Position statements on the organisation of tenders for off-patent biologicals and biosimilars have been published by these associations. Table S8 in Supplementary Materials provides an overview of the main viewpoints outlined in the position statements.

In terms of optimizing communication in the tender itself, multiple supplier interviewees mentioned that the information requested in a tender procedure should be streamlined. Only information that would be essential to the tender should be included. Continuing the digitalization of tender procedures will contribute towards increasing efficiency in this regard.

##### Healthcare Professional Involvement and Motivation

*Involving physicians in the procurement process*, avoiding top-down organized tenders, may help to increase physician adherence to the tender outcome. In Norway, the high adherence among physicians to prescribe the recommended medicine may be explained by the voluntary nature of and the involvement of stakeholders throughout the tender process [44]. Informing and educating healthcare professionals about biosimilar medicines and related concepts can also help to increase acceptance of the tender outcome.

In some countries, *benefit sharing models*—in which savings generated by tender procedures and or biosimilar use are shared between purchasing bodies or payers and the hospital—are applied to incentivize stakeholders. In England, such benefit sharing is in place between the Clinical Commissioning Groups and the trust providers. The example of the University Hospital Southampton NHS, in which a 3-year benefit sharing model was applied, reported significant cost savings and investment in clinical services (such as increasing the capacity of the nurse-led service) while maintaining similar patient-reported outcomes as result of their managed switch programme from infliximab reference product to biosimilar in inflammatory bowel patients [45].

Instead of providing a positive benefit share incentive, other approaches have been reported such as the abovementioned lowering of the reimbursement level for biological medicines for which a biosimilar alternative is available in Belgian hospitals [35].

Interviewees were in favour of organizing stakeholder incentives to increase motivation among stakeholders and support them in their work but cautioned regarding implementing rewards or quota to drive biosimilar uptake in particular. Establishing quota and incentives for the *use of best-value biologicals*, which could be either the originator or one of its biosimilars, was generally deemed more appropriate in terms of establishing a level playing field.

#### 2.2.3. Considerations Regarding the Sustainability of Tender Procedures and Their Impact on Market Dynamics

Several interviewees considered that current tender designs often focus on *maximizing short-term savings*, which they argued resulted in higher than originally anticipated price erosions. Several interviewees mentioned that the publicly reported discounts up to 70% in the Nordics established a certain precedent for subsequent price competition [23,40,46]. Although tender procedures should aim to obtain the most advantageous offer, a race to the bottom should be avoided. The majority of participants indicated that the *sustainability of current practices* should be reconsidered to ensure benefits to society and patients over the longer term.

The steep price erosion was in part attributed to the fact that companies appear to be willing to fiercely *compete on price* due to important advantages associated with winning first product volumes (“first in the market”). This would allow the supplier to gather real-world data and accustom stakeholders with their product. Early winners may also be successful in retaining the market, as the incentive to reopen soon could be low if subsequent additional savings are low and would for example not outweigh the costs (although estimated to be minimal by interviewees) and work associated with a second switch.

Moreover, originator companies appear to apply *strong defensive tactics* to maintain market share by significant price dropping. This was recognized to limit biosimilar market entry in several markets. Originator suppliers may have more leeway for pronounced discounts compared to their biosimilar counterparts due to the different stage in recuperation of development cost in the lifecycle of the product. Where biosimilar developers need to earn back biosimilar development investments upon market entry, investments are generally recouped at this stage for the originator product. Additionally, it was hypothesized that some companies lower prices to such an extent that other suppliers start to drop out. This was believed to have been the case with tender practices for adalimumab, where the originator company offered especially steep discounts in some markets. In cases where the originator swiftly dropped originator prices, originators have mostly been able to maintain a significant portion of the market.

A balance between realizing short-term savings vs. avoiding possible unintended consequences in terms of decreased competition over the mid-long term should be considered. Some markets could be more at risk than others for reduced competition, depending on the commercial opportunity in terms of volume and expected prices in the given market. Multiple suppliers believed that action is essential to prevent this evolution and cautioned that hesitations exist among developers regarding the continuation of their biosimilar programs. As counterargument, it was reasoned that not all suppliers need to remain on the market for some products, as three to four suppliers would suffice for a sustainable market environment.

In markets with high price pressure, suppliers may economize by for example reducing their emergency stock available, which adds vulnerability to the product supply chain. A race to the bottom in terms of price should be avoided. Several interviewees argued that the shortage sensitive dynamics in the off-patent small molecule market should be avoided for off-patent biologicals and biosimilars.

The development of a longer-term vision is argued to be needed, to avoid competition loss and to ensure sustainable dynamics and benefits for the healthcare system over the longer term. It was mentioned that there is a need to act now, to ensure healthcare systems and tender practices are prepared for the anticipated next wave of biosimilars reaching European markets. Collaboration between the public sector and manufacturers (umbrella organizations) is believed needed to establish such common ground and exchange of perspective. Willingness appears to exist from different parties to work towards a more sustainable framework. As several manufacturers invest in both originator and biosimilars products, consideration for sustainable tender approaches may be increasingly supported.

#### 2.2.4. Considerations Regarding Competition Dynamics—Ensuring a Level Playing Field

As noted earlier, some tender processes appear to advantage the reference product over its biosimilar (alternatives). Suppliers can attempt to steer the structuring of the tender in their favour. Competition-limiting elements (such as considering research financing) are also reported to be pro-actively requested by purchasers, which may be explained by loyalty to and (financial) ties with the incumbent. In addition to a possible deliberate steering of tender structures to favour a certain outcome/bidder, purchasers may in some cases introduce unintentional biases due to limited (procurement) expertise, questions around the structuring or hesitations regarding biosimilars. 

Examples of dynamics that favour the originator product include the delayed opening of tenders due to ongoing contracts with the originator at the time of biosimilar market entry, the application of originator favouring award criteria or offering of conditional discounts. The latter could be for example linked to the length of the contract, the ranking of the product or the offering of services that are unrelated with the subject matter of the tender, such as research financing. In the Netherlands, 20–50% of contracts were reported to include such a conditional discount structure in 2018 by a sector enquiry of the anti-Tumour Necrosis Factor product market [36,47]. Clauses that stipulate that the discounts of the competitor will be matched or renegotiated, matching the lowest offer or guaranteeing lowest price, can impact biosimilar market entry and also distort price competition, as the originator is likely to match the offer. Adding a Best and Final Offer (BAFO) round may lead to similar distortions. Contract linkage, in which offers or requests are made to provide rebates for previously delivered or contracted medicines or on a related product, in case the tender contract is won, is also reported to occur.

In case the level of price reductions offered would force biosimilar developers to compete with a price below cost of goods due to a dominant position of the originator, these can also be considered anti-competitive.

The existence of anti-competitive procurement practices warrants action. Awareness should be raised about the public procurement integrity rules; a culture of integrity should be promoted, and a better collection and analysis of data should be ensured to improve governance. Fostering the uptake of e-procurement and supporting procurers with the appropriate tools and exchange of best practice can contribute in this regard. The appropriate application of tenders should be monitored by the EU national Competition Authorities and the European Commission must support actions of EU countries in this regard [48].

#### 2.2.5. Future Outlook of Interviewees: Possible Evolutions in Tender Organization

To contain costs, competition could be further opened up by *tendering beyond the international non-proprietary name (INN)* for biologicals, which is already applied in certain cases or settings, such as in some hospital groups in the Netherlands. Including products in a same therapeutic class, which may include in some cases branded medicines, will allow to further increase competition and could be considered as option to contain spending [49].

Tender procedures may also evolve from focussing exclusively on the product’s price, to taking a more holistic approach, including the overall cost of treatment, which includes but is not limited to the medicine price. Procurement, which takes total cost of care delivery into account, also called *value-based procurement*, aims to focus on patient outcomes the product should have an impact on. Where traditional procurement may often focus on the technical specifications of the product, price and short-term benefits, value-based procurement focusses on getting a maximum patient outcome against total cost of care [50]. For instance, the total cost of the in-hospital infusion of an intravenous medicine could be compared to the cost of the patient’s self-administering of an oral medicine, or to the home-administration of a subcutaneous alternative.

Another possible foreseen development in tender practices includes *subscription-model tendering*, where for well-defined patient profiles, medicine packages focussing on the broader therapeutic needs of the patient could be tendered. Some countries and regions (US, Australia, UK) are testing such subscription-based procurement models, also called the Netflix-model [51,52]. In this type of procurement model, purchasers pay a pre-agreed flat amount to the supplier, irrespective of the volume of medicines used [51]. Such approach could provide substantial benefits as it includes a capping of costs for the payer and ‘de-risked’ revenue for the supplier. It however also increases volume uncertainty for the supplier, which could result in supply chain management challenges [53].

## 3. Discussion

Tender procedures warrant a careful organization, design, execution, and evaluation and if needed readjustment, to ensure that they are aligned with sustainable outcomes for patients, industry and society at large over the longer term. It is a delicate balancing between ensuring the most efficient use of public financial resources and safeguarding market opportunity for multiple suppliers, as such stimulating competition over the longer term. In addition to optimizing the spending of public funds, public procurement and the effective use of tender criteria beyond price may achieve other benefits for society, healthcare systems and patients [15].

In this mixed methods study, we sought to assess the experience with tendering procedures for off-patent biologicals and biosimilars in Europe and identify learnings from current practices, drawing from a quantitative web-survey in European purchasers and qualitative expert-interviews with both purchasers and suppliers.

### 3.1. Challenges in the Organization of Tenders for Off-Patent Biologics and Biosimilars

During the qualitative analysis, three main challenges arose with the organization of tenders for off-patent biological medicines and biosimilars. First, current tender practices appear to focus on realizing short-term savings. This may be explained in part by the design of the tender, which often considers only price and rewards to one single winner. Moreover, changing originator competition strategies may play a part. Whereas originator manufacturers originally appeared to protect market shares via the development of second generation or reformulated products (e.g., Humira^®^’s new formulation launch aimed for less injection pain, or the subcutaneous versions of Herceptin^®^ and MabThera^®^), strategies have shifted and include increased competition on price [2]. From the payer’s perspective, one could argue that cost savings are realized in such a scenario, regardless of any biosimilar uptake. Considering biosimilar market entry as leverage to encourage a price cut from the incumbent (via a mandated list price decrease or discounts in tender procedures) may be a successful strategy in the short-term in terms of realizing savings. However, over the mid-long term, this is likely to lead to opposite effects due to market impoverishment.

The second main identified challenge pertains to the fact that tender processes were in some cases argued to advantage the reference product over its biosimilar (alternatives). This could be both deliberately or unintentionally driven, possibly because of stakeholder preference to continue with the reference product due to brand loyalty and/or additional benefits and/or issues with the design of the tender due to limited expertise with procurement of off-patent biologicals and biosimilars. While including additional award criteria provides the opportunity to compete more sustainably on value-adding elements besides price, it also gives room for possible steering.

Third, including award criteria beyond price appears to be challenging in practice. Purchasers expressed difficulties to find the right balance between award criteria which allow to differentiate and which are non-discriminatory. From both the survey and expert-interviews, guidance appears to be needed on how to design tenders for off-patent biologicals and biosimilars and especially how to formulate appropriate award criteria. Only when truly differentiating and value-adding criteria are identified, included, objectively assessed and proportionally rewarded in the tender, the concept of MEAT can be successfully implemented and play a role in the allotment.

### 3.2. Five Main Avenues for Optimization

Based on the stakeholder insights from this study, we conclude with proposals on five identified main avenues for optimization of public procurement processes for off-patent biologicals and biosimilars: (i) safeguarding a transparent, equal opportunity setting for all suppliers; (ii) fostering a timely opening of tender procedures, ensuring on-set competition; (iii) ensuring and stimulating adherence to laws on public procurement; (iv) securing an efficient process, improving plannability and ensuring timely product supply and (v) safeguarding long-term sustainable competition. Table 4 outlines these avenues for tender optimization.

Generally, stimulating market plurality, enabling market opportunity for multiple products, is considered to be the cornerstone towards creating a more sustainable and competitive tender market by stakeholders. This is in line with the findings of the KPMG cross-country analysis into the delivery of healthcare in hospitals by optimized utilization of medicines [37] and is supported by position statements of various industry umbrella organizations (Table S8). To realize the objective of sustained product plurality, several Member States and regions have been actively pursuing new approaches from which best practices can be derived, such as the Commissioning Framework for biological medicines in England and the changes in the Italian legal framework related to biosimilars [31,54,55].

Overall, a combined action of all actors; suppliers, pharmaceutical industry associations, purchasers, payers, governments and competition authorities, is required to promote and strengthen the competition between off-patent biological medicines and biosimilars via tenders, and by extension establishing effective, healthy market dynamics. This requires a combination of practical and policy changes, involving alterations at purchaser level but also in the policy framework. Policymakers should set out a tender policy strategy, with appropriate organizational structures and stakeholder management to ensure adherence to public procurement rules. In addition to changes to the policy framework, any perverse incentives in the financing structure of purchaser bodies should be revised to ensure a level playing field. A combination of guidance (with initiatives such as horizon scanning, a tender template, award criteria framework and feedback systems), transparent reporting on the structuring and evaluation of tender procedures and monitoring and feedback from governments or competition authorities will be necessary to sensitize stakeholders in this regard.

In general, monitoring the application of the tendering policy and subsequent changes in market dynamics is warranted, together with adapting its design if needed. National authorities should actively support purchasers with the appropriate application of tender procedures and introduction of award criteria, by providing the necessary guidance and feedback. Policy makers, purchasers and pharmaceutical industry associations should take action to collaboratively develop tender frameworks that include award criteria beyond price. For example, medical devices industry associations were successful in stimulating dialogue and collaborating with contracting authorities in order to develop a methodology to encourage the uptake of value-based procurement throughout the EU [56,57].

Moreover, guidelines for biosimilar use to increase confidence and lower hurdles with the use of biosimilars [58] and an active promotion of best-value biological use by developing proper stakeholder incentivization schemes are warranted [9,55,59,60].

As exemplified by both the survey and interview data, the design and execution of tenders for off-patent biologicals and biosimilars varies across European countries, regions and hospitals. As the tender landscape is variable across Europe, measures need to be adapted to country, region and setting specific needs. The results from the expert-interviews suggest that countries in which procurement is organized on a more local or hospital individual level, where there is more flexibility and individual purchaser freedom in the design and structuring of the tender, would especially benefit from increased guidance on tender and award criteria design. European regions, where tenders are organized with a central or regionally coordinated approach, such as for example England, were generally considered to have a well thought out procurement strategy and high level of tender expertise by interviewees. In addition to a consolidation of expertise, a more central or coordinated organization of tenders aggregates purchasing needs, as such freeing up resources and time while increasing buying power.

The diverse approaches and outcomes with relation to the market entry of adalimumab biosimilars in the European countries included in the study illustrates again the diversity in healthcare systems and procurement practices across Europe. For example, NHS England and Amgros (the Danish procurement body) sought strategies to ensure rapid biosimilar adoption and generate immediate savings [54,61]. In Norway and the Netherlands, the originator manufacturer was able to retain market share by offering steep discounts [62]. Although the biosimilar market entry of adalimumab biosimilars may have been a unique casus, as a multitude of competitors were lined up to enter simultaneously the market to compete with the number one blockbuster drug worldwide, lessons can be derived, such as the importance of well in advance preparing and planning for biosimilar market entry [54,55].

### 3.3. Strengths and Limitations

The organisation of tenders for off-patent biologicals and biosimilars has been previously investigated in the context of a KPMG study on improving healthcare delivery in hospitals [37]. Here, authors identify the following elements to foster biosimilar utilization in the hospital environment: swiftly reopening of tenders, organizing multi-winner tenders, implementing benefit sharing methods and switching towards MEAT criteria. These elements are considered relatively easy to implement with a potential high impact on the system [37]. Also, the law firm Baker McKenzie performed a multi-jurisdictional European study, identifying key legal and practical aspects of the biosimilars market, in particular with regard to public tendering [63]. To the knowledge of the authors, this paper is the first scientific publication to assess in-depth stakeholder experiences with tender practices for off-patent biologicals and biosimilars and explore the sustainability of current practices.

The study presents both quantitative and qualitative data and is based on both purchaser and supplier experience. The qualitative survey data provide a snapshot of the heterogeneity of procurement practices and experiences of purchasers with the procurement of off-patent biologicals and biosimilars, across European countries. Participants from 23 different European countries, with varying levels of procurement organization (central, regional, local level) were queried. Overall, the quantitative data exemplify varying experiences across countries and provide a general overview of attitudes and challenges towards procurement of off-patent biologicals and biosimilars.

For the qualitative study part, interviews were conducted with experts in a selection of European countries that represent different tender structures, which enabled gathering information from various European contexts. In addition, interviews on a pan-European level were conducted to strengthen both country-specific and European-broad insights. Interviews were conducted with both purchaser- and supply (industry)-side participants, reflecting the insights of the two principal stakeholder groups in the tender process. The choice of qualitative interviews permitted to gain detailed insight in current practices and gather proposals for improvement from experts in the field. Experts from a purposive sample of European countries were invited, to capture a broad range of insights from countries with varying practices. However, no interview insights were obtained from Eastern-European countries. It may be useful for future research to expand on in-depth country analyses, assess perspectives of policy makers on proposed measures and conduct a systematic analysis on tenders in the EU database on tenders.

The general set of principles and proposals as outlined in Table 4, based on pan-European and country specific expert insights, could be applied mutatis mutandis to specific countries and settings. It is important to note that not all findings are generalizable to the whole off-patent biologicals segment across Europe, as some are product, country, setting or time related. Depending on the tender organization and maturity of the respective country or setting, measures on different levels may be needed and these should be tailored to country context. Some of the proposed recommendations are based on existing best practices. Several countries, regions or hospitals implement at present one or multiple of the proposed practices as outlined in Table 4. Some learnings may not be limited to tender practices for originator biologicals and biosimilar and might apply to tender practices in general. The fact that discounts in tender procedures are generally confidential prevents to properly mirror the gathered qualitative insights on price competition with actual price data beyond list-price level. As estimated by IQVIA, confidential discounts range between 10% and 90% on list price and could offer a 5–10% saving to the overall drug budget [8].

**Table 4:** Proposals on how to optimize tender procedures for off-patent biologicals and biosimilars, ensuring sustainable competition and associated savings in the long-term in the off-patent biologicals segment—five main avenues for optimization.

Tender practices should abide with the European Union and Member State rules on tendering. The involved actors, suppliers, purchaser bodies, payers, government and competition authorities, have a role to fulfil to ensure efficient, fair and transparent tender procedures for off-patent biologics and biosimilars. - Purchasers (hospital or procurement body): securing a transparent and efficiently managed process - Industry/suppliers: ensuring timely, non-disrupted and high-quality supply - Payers: establishing adequate incentives and resolving any counterproductive motivational schemes Government: enabling sustainable market competition, by implementing policies and tender structures with a long-term perspective. Stimulating market plurality and providing guidance to purchasers - Competition Authority: monitoring the correct application of tenders, by performing audits and following up purchaser adherence with laws on public procurement The proposals outlined below can be considered as a general set of principles that can inform the different actors involved on possible improvements. Depending on the tender organization and maturity of the respective country or setting, measures should be selected and tailored to the country’s context. Some of the proposed recommendations are based on existing best practices. Several countries, regions or hospitals have implemented already one or multiple of the proposed practices as outlined here.
**1.** **Safeguarding a transparent, equal opportunity setting for all suppliers, with an appropriate use of award criteria**
The tender procedure needs to be **transparent** and **non-discriminatory** with **predefined rules and pathway**, which are **adhered to** throughout the process - Contracts should be awarded on the basis of **objective criteria that are compliant with the principles of transparency, non-discrimination and equal treatment** (as stipulated in the EU Directive (§ 90)) [13], allowing an objective comparative assessment. - **Other award criteria besides price** that add value to the contract should be included, **applying the Most Economically Advantageous Tender (MEAT)** procedure as stimulated in the EU Public Procurement Directive [13], avoiding lowest bid procedures and stimulating suppliers to compete sustainably on more criteria - **A clear framework regarding selection—and award criteria** should be implemented and adhered to: ○ Selection and award criteria should be carefully formulated, to avoid that participants are excluded a priori or certain products are disadvantaged on improper grounds. **Criteria for which longer market presence is required or would be advantageous should be avoided,** as these could lead to unreasonable competition expulsion, disadvantaging recently launched products. ○ Only criteria that are **related and proportionate** to the subject matter should be included. Any criteria that could unreasonably limit competition or introduce bias should be excluded. The link with the subject matter should be clear. Caution should be exerted regarding requesting or offering additional services or benefits, and this should be strictly avoided if not directly related to the subject matter. ○ Only additional criteria that **drive actual benefits** (meaningful product differentiation, advantage for purchaser and/or patient) should be included. ○ Award criteria besides price should be **proportionally rewarded** based on the additional value created, as the provision of additional services increases investment for suppliers. This will also enable these criteria to truly play a role in the allotment. ○ **Relevant experts** should be integrated to identify appropriate award criteria. In countries where procurement is organized on a local or individual hospital level, **governments** should provide **guidance** to purchasers regarding the structuring of the tender and application of selection and award criteria. Here, a **flexible/semi-structured tender template** could be designed to guide purchasers but also allowing room for tailoring based on product—specific considerations and strategic differentiation. - Contracting authorities should **timely and transparently inform** possible competitors about the criteria that will be applied in the contract, by **specifying the award criteria** as well as **the relative weight or the allocation of points** given to each of those criteria in advance. - **Linkage between contracts** (e.g., offer of or request to supplier to provide rebates for previously delivered or contracted medicines or rebates on a related product, in case the tender contract is won) can impact the equal opportunity setting and limit competition. Offering extensive conditional rebates with dominant position of the originator can be considered as anti-competitive exclusion. - Avenues that provide **anonymity throughout the procedure**, such as requesting that bids are filed anonymously with coding identifier, should be applied where possible to avoid incumbent advantages [64]. - In the case of **preliminary market consultations,** these should guarantee that every supplier is **treated equally** and receives the **same information.** Although dialogue between purchasers and suppliers should generally be fostered to improve understanding of each other’s needs on an overarching level, no direct input should be sought on the structuring of the tender from a supplier, as this could introduce steering of the structure of the tender.
**2.** **Fostering a timely opening of tender procedures, ensuring on-set competition**
Tender procedures should be **opened as soon as possible**, to avoid delays in competition and market opportunity for biosimilar competitors: - Tender procedures should be prepared to **timely open:** ○ Systems should be prepared to organize tenders upon biosimilar market entry to reduce barriers to entry. A continuous re-opening of procedures with every new competitor entering the market should however be avoided, as this could introduce uncertainty in terms of volume and tender duration for the first tender winner(s) (lowering volume predictability) and also be burdensome for contracting authorities and industry. ○ Installing a shorter-term tender (e.g., 6 months) immediately upon market entry of the first biosimilar competitor(s), combined with a longer subsequent tender agreement, would allow immediate competition and market opportunity for the different competitors once the market has further crystalized in terms of number of available products. ○ Alternatively, a differentiated, product—specific approach in determining the appropriate term for opening a tender, taking into account the number of expected competitors, could be appropriate. - **A specialist procurement office** involving the appropriate expertise fields could play an important role in organizing and coordinating the timing and duration of tender procedures for products with biosimilar competition. Moreover, such expert coordination office, should apply a long-term view, taking future biosimilar market entry into account to advice on negotiated contract duration, avoiding **blocking contracts** at the time of biosimilar market entry. Such an expert procurement office should perform **horizon scanning** to identify the upcoming loss of exclusivity of reference products and anticipated biosimilar market entry dates. (cfr. infra, bullet D). Such expert procurement office or payers could also strategically set out incentive schemes to stimulate a timely opening of procedures, as needed. - A **financial stimulus** should be put in place to stimulate purchasing bodies/hospitals to organize tenders, aligning the incentives of the purchaser with these of the overall healthcare system (savings for healthcare budgets). - A tender **duration between 12 and maximum 24 months** would be desirable to stimulate market dynamics, while considering feasibility and avoiding frequent switching.
**3.** **Ensuring and stimulating adherence to laws on public procurement**
The rules on public procurement should be correctly applied: - **Competition authorities should monitor and audit** the correct, timely and transparent implementation of and adherence to the laws on public procurement by purchasers and **investigate signals of anti-competitive conduct** (e.g., conditional rebates). If needed, they should take **appropriate measures**, ensuring a timely opening of tenders and the application of appropriate award criteria. - Governments should provide **feedback** to purchasing bodies **on performance** and apply **steering measures** where needed. - For decentralized purchasing systems, the route of establishing a dedicated, independent and centrally coordinated expert panel (involving lawyers, physicians, pharmacists), to conduct the assessment, could be explored. The transferring of assessment to an independent central organ could improve objectivity of and ensure the appropriate expertise in the evaluation. - Stakeholders should be stimulated to **actively report** any signals of anti-competitive conduct to the competition authority. - **Financing streams/structures** of purchaser bodies and involved stakeholders should be **reviewed, removing existing disincentives** and **introducing new incentives** that are aligned with the overall healthcare system ○ **Disincentives** to organize competitive tenders or **incentives that favour a specific product/preference for the originator/more expensive product** should be **removed.** ○ **Financial incentives schemes or other policies** should be put in place: ▪ Top-down: such as **lowering the reimbursement level** of products that are open to competition to stimulate purchasers to timely organize competitive tender procedures. ▪ Stakeholder-involved: Savings from tender procedures could be allocated in part to remunerate HCPs for their time investment in switching, as part of a **gain-sharing model**. Such a gain-sharing model could motivate and involve stakeholders, increasing adherence to the tendered winner(s) and countering possible financial incentives and preferences to use the originator product. - In addition to motivating stakeholders via above mentioned incentive schemes, **multi-winner tenders** or tenders with a ranking of preferred products can help to increase physician adherence to the tender outcome (avoiding physicians’ use of the higher priced non-preferred product), as it may increase **physicians’ freedom** to choose between available products**. Involving physicians in the tender procedures,** e.g., in the Drug & Therapeutic committee is also considered important in this regard. - Authorities and governments should also support stakeholders with **up-to-date guidelines for biosimilar use** (e.g., on (multiple) switching) and develop **policies and information campaigns to improve stakeholder confidence** in biosimilars and **increase awareness on their benefits**. This may help lowering practical barriers associated with biosimilar use and uncertainty among stakeholders.
**4.** **Securing an efficient process, improving plannability and ensuring timely product supply**
The tender procedure needs to be efficiently managed, optimizing and reducing the administrative and time burden for both suppliers and purchasers, as well as increasing predictability and plannability for the supplier—supporting timely product supply. - The **predictability and plannability of tender procedures and** associated **volumes** to be supplied should be **improved** towards suppliers**:** ○ This includes setting of **reliable estimates of volume to be supplied** (with guaranteeing minimum volumes and a maximum cap), **timely communication regarding** the **timing** of tender procedures and making use of **acceptable lead times** to support suppliers to better forecast and anticipate on demand, as such reducing the risk of shortages. ○ In case of supply issues, **penalties** should be **proportionate** to the contract value and the cause of the inability to supply (force majeure/external reasons for which could not be controlled), ensuring a fair balance of risk and reward. In case of inability to supply volumes that are higher than estimated (e.g., not specified in procurement contract), the supplier should bear no (disproportionate) financial risk. ○ In some cases, a good strategy could be that tender procedures **open up throughout the year**, to spread commercial opportunity for suppliers and accommodate manufacturing capacity. - **Expertise on procurement** should be **consolidated** to actively **guide purchasers** in **timely and efficiently** setting up tender procedures ○ A **dedicated, expert procurement office** that consolidates knowledge, skill and experience with tender procedures should be available **to support purchasers/procurement bodies with the timely planning and efficient organization** of tender procedures. ○ Such a specialized procurement office should **set out strategy**, **coordinate** purchasers and tender procedures, **perform horizon scanning** to inform stakeholders on the upcoming loss of exclusivity of reference products and anticipated biosimilar market entry dates, **prepare stakeholder guidance** documents and **monitor** the number of competitors on the market. ○ Beyond coordinating the procurement strategy for products with anticipated biosimilar competition, the expert office should apply a long-term view and advice on contract length of new contracts, **considering future market entries**, **avoiding “blocking” contracts** at the time of biosimilar market entry, which would delay market competition. ○ **Specific measures** or a **tailored approach** could be applied to prepare for biosimilar market entry of a specific product (or product category) (as was done in several countries to prepare for adalimumab biosimilar market entry) or could be adjusted based on specific market dynamics. ○ Such an overarching expert office would also be beneficial in terms of **consolidating efforts, avoiding duplication** and **professionalising** the processes, as required by the increasingly complex structure of tender procedures. ○ Depending on the country, such expert centre could be established **at national or regional level**. - **Tender procedures and documentation requests should be harmonized, simplified and made leaner** to mitigate the administrative workload and increase efficiency, also reducing the possible sunk cost of participating suppliers. E-procurement should be wider used to allow information to be easily accessible throughout the tender procedure for both purchasers and suppliers. Beyond reducing the administrative burden, this will allow a higher traceability and transparency of procedures [24]. The process should also be streamlined in terms of the **information** which is believed to be **essential**. **Operating on a larger scale** by grouping purchaser bodies and the existence of an **expert procurement office** guiding procedures could benefit purchasers and suppliers in this regard.
**5.** **Safeguarding long-term sustainable competition by stimulating market plurality**
The tender procedures and overall procurement strategy need to take a long-term view into account, tailored to supporting long-term sustainability, providing commercial opportunity for multiple suppliers - **Stimulating market plurality and multiple commercial opportunities for suppliers** ○ Single-winner tenders can exclude non-winning competitors from the market for the duration for the tender contract, and long-term lead to reduced levels of competition. **Ensuring market plurality is a cornerstone for a sustainable and competitive tender market and should be part of tendering strategy.** Depending on market size and specific context (product volume), different scenarios can be appropriate and applied. **Multi-winner tender** can be organized on national level or regional level (if there is a **sufficiently large scale**), or markets could be divided into **multiple commercial single-winner opportunities** (e.g., on hospital network or regional level). ○ In the case of the scenario of multiple single or multi-winner opportunities across the market, a **rotating system** between regions or hospitals could be set up to increase dynamics and opening of commercial opportunities for suppliers over time. ○ Multi-winner tenders also provide price reductions on all tendered products and may increase the physician’s **therapeutic freedom** to choose between different products, as such avoiding physicians using a higher-priced non-preferred product. ○ The availability of multiple commercial products on the market may also help to mitigate possible **supply issues**. - **Regular evaluation of the market situation and if needed revision of procurement and tendering mechanisms** ○ Market dynamics such as the numbers of competitors and associated manufacturers should be reviewed on an annual basis and tendering policies should be reviewed in this context, avoiding market concentration and de novo monopolies

### 3.4. Balancing Short and Long Term Benefits

It is clear from this study that it is a delicate balance between optimizing efficient spending of public funds, addressing patient needs and preserving competition over the longer term. When designed efficiently and conducted appropriately, tenders can stimulate competition and as such form a cornerstone for sustainable market dynamics. As ensuring the most efficient use of public resources and broad access to medicines is a common societal goal, actions to ensure that tender processes are effective and motivate suppliers to participate over the longer-term are essential. Starting in the next five years, the number of biologic loss of exclusivities will increase substantially [8]. Healthcare systems across Europe need to be prepared to facilitate and optimize market access for and competition from the next wave of biosimilar market entries, drawing from earlier experiences. This will allow healthcare systems to maximize the benefit of biological competition efficiently over the long term.

## 4. Materials and Methods

The study follows a mixed methods design, consisting of a survey and semi-structured interviews, gathering both quantitative and qualitative data. The study concentrates on tender procedures organised by contracting authorities. Tenders that are organized by private entities are not bound to organise public procurement procedures and are therefore out of scope. Ethics approval of the study was granted by the Research Ethics Committee UZ/KU Leuven (MP006498, Belgium).

### 4.1. Quantitative Web-Survey

#### 4.1.1. Recruitment

A quantitative, anonymous web-questionnaire was developed to survey purchasers and hospital pharmacists about the organisation of tenders for off-patent biologicals and biosimilars. The survey was disseminated to hospital pharmacists and purchasers across Europe, via professional associations such as the European Association of Hospital Pharmacists, by contacting procurement entities and the network of the research group.

#### 4.1.2. Survey Development

The survey was developed based on a study of the literature and consisted of questions about (i) the experience of participants with tender procedures for off-patent biologicals and biosimilars and perceived challenges, (ii) the design of tender procedures (number of winners, average tender duration, reopening of tenders, physician involvement), (iii) the application of selection-and award criteria and (iv) considerations about interchangeability and switching, as tenders may result in an exchange of products. The survey questions were refined based on comments from both a hospital pharmacist and a supplier. The survey was developed online in the KU Leuven Websurvey-server and gathered anonymous data. The survey consisted of closed multiple choice, ranking or Likert-scale questions. Participants were given the possibility to add additional information in an open text field for certain questions and answer options such as “Other”. The first window of the web survey provided participants with information about the study, the voluntary nature of participation and a statement regarding agreement to participate. The survey was anonymous, and no personal data were collected.

#### 4.1.3. Analysis

Responses were gathered between October 2018 and February 2019. The survey answers were analysed descriptively on an overall group level.

### 4.2. Qualitative Semi-Structured Interviews 

#### 4.2.1. Recruitment

To gather qualitative, in-depth expert insights regarding the organization of tenders for off-patent biologicals and biosimilars, semi-structured interviews were conducted with hospital pharmacists, purchasers and pharmaceutical industry employees. The sampling was purposeful to obtain a range of experiences and perspectives, reflecting both the purchaser and supply side perspective, from individuals that are knowledgeable about and experienced with tender processes for off-patent biologicals and biosimilars.

Eligible participants worked currently or formerly as (i) medicine purchaser or hospital pharmacist, (ii) in, or as consultant to, a pharmaceutical company with at least one EMA-approved originator biological or biosimilar (or having both originator and biosimilar products) or for a pharmaceutical industry trade organization. Employees from both legacy originator and legacy generic companies were recruited. Participants were selected for their experience with and knowledge about tender practices for off-patent biologics and biosimilars [65].

To capture diverse and comprehensive insights, both participants with insights on a pan-European level (e.g., from European professional associations, European pharmaceutical company headquarters or trade organizations) and participants with country specific insights were invited. For the latter, participants were recruited from a purposive selection of seven European countries, representing different tender organizational systems (central purchasing: Denmark and Norway, regional purchasing: England and Italy, buying group/hospital individual purchasing: France, the Netherlands and Belgium). The choice to capture the insights of both purchaser- and supply (industry)-side participants was made to obtain views from the two principal stakeholder groups in the tender process.

Participant recruitment was carried out by screening relevant websites, scientific and professional stakeholder associations, relevant conferences and publications and the network of the research group for eligible participants.

While different sampling strategies were applied for the survey and the interviews (broad vs. purposeful sampling), a certain overlap in participants may theoretically have been possible. The impact of having a respondent possibly participating in the survey and a subsequent interview is considered negligible on interview results since the survey and interviews served distinct purposes.

#### 4.2.2. Interview Guide and Interviews

Interviews were carried out in English, with the exception of a few interviews in Dutch, in person, via telephone or teleconference between March 2019 and February 2020. All participants provided written informed consent prior to the start of their interview. Consent was given by all participants for using the encoded and anonymized data from their interview for scientific publication. Interviews were conducted using an interview guide based on topics identified from scientific literature, policy documents, position statements related to the procurement of off-patent biologics and biosimilars and the quantitative survey results. Interviewees were asked to share their insights on challenges, best practices and learnings regarding tender practices for off-patent biologicals and biosimilars, as well as proposals towards long-term sustainable tender practices. An overview of discussed topics is shown in Supplementary Materials Table S9. All interviews were audio-recorded and transcribed verbatim. Interviews were carried out until saturation of the data [65].

#### 4.2.3. Analysis

Interview transcripts were pseudonymised and analysed according to the thematic framework method, using Nvivo^®^ data analysis software [66].

## 5. Conclusions

This study found that opportunity exists to improve tender practices for off-patent biologicals and biosimilars in Europe. In order to realise the competition potential of biosimilars and benefits from appropriate tender procedures for healthcare systems and patients, concerted actions by policymakers and purchasers, in dialogue with industry associations, with a long-term strategic view are needed to optimize tender frameworks. Depending on the country’s policy environment and the maturity of the procurement body, different sets of policy and practical measures are needed. In general, measures should aim to ensure supplier market plurality, establish a transparent and objective process, and include award criteria beyond price. This may contribute to creating a sustainable climate, with long-term competition in the off-patent biologicals market.

## Figures and Tables

**Table 1:** Highlights.

**What is already known about the topic?**
The organization and implementation of tendering of off-patent biologicals and biosimilars varies across European Member States, regions and purchasing groups. The organization and design of tenders may play a key role in the evolving biosimilar market. It is not fully elucidated how tenders for off-patent biologicals and biosimilars are designed, and if approaches are aligned with sustaining market competition and societal savings for healthcare systems over the long term.
**What does the paper add to existing knowledge?**
This mixed methods study reports quantitative results derived from a survey among purchasers and hospital pharmacists regarding the application of tenders and qualitative insights from expert-interviews with suppliers and purchasers. This paper puts forth an actionable framework with proposals that could contribute towards a more sustainable organization and application of tenders for off-patent biological medicines and biosimilars in Europe.
**What insights does the paper provide for informing health care-related decision-making**
Findings may inform and support purchasers, suppliers and policymakers regarding the organization and optimization of tender procedures for off-patent biologicals and biosimilars. Tender procedures must aim to (i) ensure market plurality and (ii) include award criteria beyond price (warranted that criteria are objectively and transparently defined, scored and competitively rewarded). Depending on the market (maturity), additional actions are considered needed.

In this manuscript, the term “off-patent biologicals” refers to reference biologicals that lost patent protection and are exposed to competition from biosimilar alternatives.

**Table 2:** Organizing multi-winner tenders—considerations.

Advantages	Conditions
Stimulating market presence of multiple suppliers over the longer term	Volume at purchaser level needs to be sufficiently large to be divided among different suppliers. Alternatively, multiple single-winner opportunities can be organized in a given market to ensure supplier plurality (i.e., the approach and number of winners should be adjusted to market purchasing characteristics.)
Offering commercial opportunity to multiple suppliers	The purchaser’s capacity needs to be sufficiently consolidated to accommodate the increased complexity and workload
Lowering immediate steep price pressure (avoid one winner takes all), which may lead to more sustainable price dynamics over the longer term	The allocation of volume between suppliers needs to be clear and guaranteed
Providing price reductions on all tendered products	
Possibly increasing physician’s product choice	
The availability of multiple commercial products on the market may help to mitigate supply issues in case shortages would occur	

**Table 3:** A selection of criteria to consider and avoid in tender procedures.

**A Selection of Possible Criteria to Consider beyond Price**
**1.** **Quality and technical related criteria** Presentation: vial size, available concentrations/dosages strengths, vial protection, etc. Packaging: labelling, storage volume, etc. Storage conditions: shelf life, stability pre-post-reconstitution, stability in/out of refrigeration, etc. Reconstitution and product administration: reconstitution time, efficient use/handling, e.g., ready to use formulation, pre-filled syringe, etc. Indications: authorization and reimbursement status
**2.** **Service-related criteria** Supply: (number of) manufacturing, packing and storage location(s), logistics arrangements, urgent delivery modalities, customer support, policy on returns/expired products, policy on strategic stocks Value added services related to the subject matter: home delivery, nurse service at home, therapeutic drug monitoring support, training and educational support for HCPs, etc. Environmental and sustainability criteria: sustainability/environmental company policy (production, transport), sustainability/environmental policy of subcontractors, packaging material
**3.** **Patient related criteria** Product administration: (easiness of use of) device, injection pain (needle size, buffer, volume, etc.) Patient-driven services related to the subject matter: patient support program (online disease education, device training, adherence program, etc.), patient information material
**A Selection of Less Desirable Criteria to Consider**
*Only criteria that drive actual benefits (meaningful product differentiation, advantage for purchaser and/or patient) and are related to the subject matter should be included. The below criteria may be considered to impact the level playing field between products, to be misaligned with the biosimilarity principle and/or to be of limited value.*
**1.** Criteria that require the product **to be already on the market for a certain period of time**, as these would naturally advantage products with longer market presence, i.e., the originator product, and disadvantage recently launched biosimilars
E.g., requiring product sales references of the previous 3 years **2.** **Broad application of benefits or extra services** that are **not directly related to the subject matter** E.g., financial resources/grants for research or financial support to attend conferences or trainings
**3.** Award criteria related to the **efficacy, safety or quality profile of the biosimilar product** EMA evaluates the biosimilar candidate, once licensed there is no need to reassess the work of the regulator. Criteria should be formulated based on a full understanding of the biosimilarity principle (e.g., rewards on the extensiveness of the clinical development, although these might be convincing for clinicians, are less desirable).
**4.** Request for **clinical switch data** or financial support to conduct a **switch study** This would generate an additional evidence generation hurdle beyond biosimilar licensing requirements The national competent authority provides guidance in this regard **5.** **Contract linkage** via **conditional discount offerings** or other price structuring beyond product price could limit competition E.g., between linkage between SC and IV products, where only the IV segment is open to biosimilar competition

EMA: European Medicines Agency, HCPs: healthcare professionals, IV: intravenous, SC: subcutaneous. Consulted reference materials, besides interview transcripts: tender contracts, [29,30].

## Data Availability

The survey data presented in this study are available on reasonable request from the corresponding author. The interview data are not available upon request as they contain information that could compromise interviewees’ privacy and consent.

## Supplementary Materials

The following are available online at https://www.mdpi.com/article/10.3390/ph14060499/s1, Table S1: Survey participants’ characteristics, Table S2: Experience with and perceptions about tenders for off-patent biologicals and biosimilars, Table S3: Structuring of tenders for off-patent biologicals and biosimilars, Table S4: Application of selection and award criteria in tenders for off-patent biologicals and biosimilars, Table S5: Interchangeability and switching considerations in tender design, Table S6: Interview participants’ characteristics, Table S7: Interview participants’ characteristics, Table S8: Recommendations extracted from position/white papers on tender procedures (for off-patent biological medicines and biosimilars) in Europe, Table S9: Main topics addressed during the interviews.
