# Supplementary material for: Off-Patent Biologicals and Biosimilars Tendering in Europe—A Proposal towards More Sustainable Practices
Source: Pharmaceuticals (Basel). 2021 May 24;14(6):499. doi: 10.3390/ph14060499 (PMC8225063; doi:10.3390/ph14060499)
Supplement: Supplementary file 1 [file pharmaceuticals-14-00499-s001.zip › Supp Table S1.pdf]

Off-patent biologicals and biosimilars tendering in Europe – a proposal towards more sustainable practices

Pharmaceuticals – Biosimilars special issue

Liese Barbier, Steven Simoens, Caroline Soontjens, Barbara Claus, Arnold G. Vulto, Isabelle Huys

Corresponding author: Liese Barbier, Email: liese.barbier@kuleuven.be

**Supp. Table S1. Survey participants' characteristics**			
**Characteristics**	**Participants**		**N =**
	**N**	**%**	
**In which country do you work?**			60
Belgium	11	18	
Bulgaria	3	5	
Croatia	1	2	
Cyprus	1	2	
Czech Republic	1	2	
Denmark	1	2	
France	2	3	
Finland	2	3	
Germany	1	2	
Ireland	1	2	
Italy	1	2	
Latvia	1	2	
Malta	3	5	
Netherlands	2	3	
Norway	5	8	
Poland	3	5	
Portugal	7	12	
Romania	3	5	
Slovakia	3	5	
Slovenia	1	2	
Spain	4	7	
Sweden	1	2	
UK	2	3	
**In which type of institution do you work?** *Multiple answers possible*			60
Hospital pharmacy	40	67	
Hospital procurement unit	4	7	
Health insurance company	1	2	
National purchasing group	4	7	
3rd party payer	1	2	
Regional purchasing group	5	8	
National healthcare authority/Ministry of health	6	10	
Public health service	1	2	
University	12	20	
Other	5	8	
**Please indicate if you work in a(n)…***			42
Academic hospital	18	43	

Regional hospital	20	48	
Other	4	10	
**Please indicate if you work in a(n)…***			42
Public hospital	34	81	
Private hospital	6	14	
Other	2	5	
**For participants who work in a hospital*			
Abbreviations: N: number			
