# Supplementary material for: Off-Patent Biologicals and Biosimilars Tendering in Europe—A Proposal towards More Sustainable Practices
Source: Pharmaceuticals (Basel). 2021 May 24;14(6):499. doi: 10.3390/ph14060499 (PMC8225063; doi:10.3390/ph14060499)
Supplement: Supplementary file 1 [file pharmaceuticals-14-00499-s001.zip › Supp Table S2.pdf]

Off-patent biologicals and biosimilars tendering in Europe – a proposal towards more sustainable practices

Pharmaceuticals – Biosimilars special issue

Liese Barbier, Steven Simoens, Caroline Soontjens, Barbara Claus, Arnold G. Vulto, Isabelle Huys

Corresponding author: Liese Barbier, Email: liese.barbier@kuleuven.be

Supp. Table S2. Survey questions and results - Experience with and perceptions about tenders for off-patent biologicals and biosimilars

Question	Answer options	Results
What is the experience of your organisation with tendering for biological medicines?	Not (yet) tendered for biological medicines	7% (4/56)
	Limited experience	32% (18/56)
	Moderate experience (conducted multiple tenders for biologicals)	36% (20/56)
	Extensive experience	25% (14/56)
Please indicate which stakeholders participate in formulating the tender conditions/criteria and subsequent product selection: *(multiple answers possible)*	Hospital pharmacist	88% (50/57)
	Physician	68% (39/57)
	Procurement office	67% (38/57)
	Hospital board of directors	26% (15/57)
	3rd party payer	3% (2/57)
	Insurer	2% (1/57)
	Other	25% (14/57)
	Mentioned open answers under "Other": hospital management, lawyer, nursing staff, patient representative/organization	
Are there differences between tendering procedures applied for biologicals and small molecule medicines?	Yes	44% (23/52)
	No	46% (24/52)
	I don't know	8% (4/52)
	Other	2% (1/52)
	Mentioned differences under "Yes": more attention towards discussion with prescribers, greater involvement of HCP staff due to switch, reimbursement, deciding to include biosimilar and RP in same lot or not, switching restrictions, administration route, device or dosage, differences in internal mandate; the emotion with biologicals is greater, tender level: on active substance vs therapeutic group, criteria applied	
	Mentioned open answer under "Other": theoretically not, but there are difficulties with switching patients	
Which challenges do you identify with tendering for biological medicines?	Questions about interchangeability and switching between a biological	60% (31/52)

(multiple answers possible)	reference product and its biosimilar(s)	
	Formulation of award criteria	25% (13/52)
	Supply chain reliability	23% (12/52)
	I do not identify specific challenges with tendering for biological medicines, different from those with tendering for medicines in general	21% (11/52)
	Formulation of criteria to select viable suppliers	19% (10/52)
	Other	10% (5/52)
	Mentioned open answers under "Other": knowledge of involved stakeholders, switching between biosimilars, device	
Abbreviations: RP: reference product		
