# Supplementary material for: Off-Patent Biologicals and Biosimilars Tendering in Europe—A Proposal towards More Sustainable Practices
Source: Pharmaceuticals (Basel). 2021 May 24;14(6):499. doi: 10.3390/ph14060499 (PMC8225063; doi:10.3390/ph14060499)
Supplement: Supplementary file 1 [file pharmaceuticals-14-00499-s001.zip › Supp Table S3.pdf]

Off-patent biologicals and biosimilars tendering in Europe – a proposal towards more sustainable practices

Pharmaceuticals – Biosimilars special issue

Liese Barbier, Steven Simoens, Caroline Soontjens, Barbara Claus, Arnold G. Vulto, Isabelle Huys

Corresponding author: Liese Barbier, Email: liese.barbier@kuleuven.be

Supp. Table S3. Survey questions and results - Structuring of tenders for off-patent biologicals and biosimilars

Question	Answer options	Results
What is the average tender duration?	< 6 months	12.5% (7/56)
	6 months – 1 year	21% (12/56)
	> 1–2 years	27% (15/56)
	> 2–3 years	20% (11/56)
	> 3–4 years	12.5% (7/56)
	> 4 years	0% (0/56)
	Irregular duration of tenders	0% (0/56)
	Other	7% (4/56)
	Mentioned open answers under "Other": there can be exceptions	
Can a contract/tender be reopened after loss of exclusivity of the tendered originator product?	Yes	55% (31/56)
	No	29% (16/56)
	Other	16% (9/56)
	Mentioned open answers regarding timing of reopening under "Yes": depends on workload, depends on national P&R procedure, depends on the product, between 0-6 months, within 3 months, within 12 months, instant	
	Mentioned open answers under "Other": depends on the original contract, depends if the product is on the market, a second tender can be opened in parallel, not done so far, unsure	
When tendering for biological medicines:	Tenders are generally awarded to a single winner	46% (21/46)
	Both single and multiple winner constructs are possible, depending on the product (class)	46% (21/46)
	Tenders are generally awarded to multiple winners	9% (4/46)
	Mentioned in open answer field for explanatory info: depends on the clinicians' request, depends on the reliability of supply (multiple products to prevent shortages), if switch is insufficiently documented multiple winners are selected including the previous product, multiple winners to ensure market sustainability, depends	

	on the approved indications (all the needed indications need to be covered), multiple winner in case of more than 3 biologicals are available with the same INN, administration route and dosage, ranking of multiple products according to price, recommendations are made based on lowest annual cost, if the winner can't honour the order, the order goes to the second winner	
How is the physician's prescribing decision incorporated when tendering? *(multiple answers possible)*	Physicians can request a motivated exception to prescribe a different product than the tendered product	68% (39/57)
	Physicians are represented in the prescribing/tendering committee	56% (32/57)
	Physicians needs to prescribe the tendered winner	53% (30/57)
	Physicians maintain the therapeutic freedom to prescribe a different product than the tendered product	10.5% (6/57)
	Other	12% (7/57)
	Mentioned open answers under "Other": physicians can deviate from tendered product for a certain percentage of patients, motivated exceptions are vetted and decided upon by an independent committee	
Abbreviations: INN: international non-proprietary name, P&R: pricing and reimbursement		
