# Supplementary material for: Off-Patent Biologicals and Biosimilars Tendering in Europe—A Proposal towards More Sustainable Practices
Source: Pharmaceuticals (Basel). 2021 May 24;14(6):499. doi: 10.3390/ph14060499 (PMC8225063; doi:10.3390/ph14060499)
Supplement: Supplementary file 1 [file pharmaceuticals-14-00499-s001.zip › Supp Table S4.pdf]

Off-patent biologicals and biosimilars tendering in Europe – a proposal towards more sustainable practices

Pharmaceuticals – Biosimilars special issue

Liese Barbier, Steven Simoens, Caroline Soontjens, Barbara Claus, Arnold G. Vulto, Isabelle Huys

Corresponding author: Liese Barbier, Email: liese.barbier@kuleuven.be

**Supp. Table S4. Survey questions and results - Application of selection- and award criteria in tenders for off-patent biologicals and biosimilars**		
What is the relative weight that is given to price when tendering for biological medicines?	Predominately on price	38% (18/47)
	100% on price	23% (11/47)
	50% on price, 50% on other criteria	19% (9/47)
	Predominantly on other criteria besides price	19% (9/47)
How are the selection and award criteria formulated? *(multiple answers were possible)*	In collaboration with/with advice from experts within the own organization	70% (33/47)
	Based on previous tender experiences	53% (24/47)
	Based on national or European guidelines	43% (20/47)
	In collaboration with/with advice from (one of) the suppliers	13% (6/47)
Are there differences between the selection criteria for suppliers applied in tender procedures for small molecules and these for biological medicines?	Yes	22% (11/50)
	No	68% (34/50)
	I don’t know	10% (5/50)
	Mentioned differences under “Yes”: interchangeability data/switching studies are requested, support with implementation is needed, for biologicals only price, higher requirements regarding reliability of supply as we cannot interchange	
Which selection criteria are applied to select viable suppliers of biological medicines? *(multiple answers were possible)*	The financial viability of the supplier	27% (14/51)
	The supplier’s production capacity	20% (10/51)
	The supplier’s reputation	20% (10/51)
	The supplier’s track record of previous tenders	16% (8/51)
	Previous collaboration with the supplier	12% (6/51)

	The duration that the supplier already markets the product	8% (4/51)
	The supplier's investment in R&D	8% (4/51)
	The product's market share	6% (3/51)
	The supplier's investment in academic research	4% (2/51)
	The supplier's investment in clinical trials	2% (1/51)
	Not applicable: no selection criteria for the supplier since we conduct open tenders	39% (20/51)
Are there differences between the award criteria applied in tender procedures for small molecules and these for biological medicines?	Yes	33% (16/48)
	No	60% (29/48)
	I don't know	6% (3/48)
	Mentioned differences under "Yes": depends on the product, can be due to patented indications, switch studies, different weight on other criteria vs price, separate lot for patients under treatment and new patients	
What are the award criteria that are applied to select the winning biological product(s)? *(multiple answers were possible)*	The product's registered indications	49% (24/49)
	The product's stability/shelf life	45% (22/49)
	The supply conditions	41% (20/49)
	The delivery device	35% (17/49)
	The packaging	35% (17/49)
	Emergency delivery and 24/7 reachability supplier	29% (14/49)
	Additional efficacy and/or safety data (in addition to data for regulatory approval)	22% (11/49)
	Value added services (e.g. supporting educational activities, product training programs, information brochures for HCPs or patients about the product, support with switching from the medicinal product previously used)	18% (9/49)
	Customer support	14% (7/49)
	Expenses incurred from switching from the previous winner	6% (3/49)
Abbreviations: HCPs: healthcare professionals; R&D: research and development		
