# Supplementary material for: Off-Patent Biologicals and Biosimilars Tendering in Europe—A Proposal towards More Sustainable Practices
Source: Pharmaceuticals (Basel). 2021 May 24;14(6):499. doi: 10.3390/ph14060499 (PMC8225063; doi:10.3390/ph14060499)
Supplement: Supplementary file 1 [file pharmaceuticals-14-00499-s001.zip › Supp Table S5.pdf]

Off-patent biologicals and biosimilars tendering in Europe – a proposal towards more sustainable practices

Pharmaceuticals – Biosimilars special issue

Liese Barbier, Steven Simoens, Caroline Soontjens, Barbara Claus, Arnold G. Vulto, Isabelle Huys

Corresponding author: Liese Barbier, Email: liese.barbier@kuleuven.be

**Supp. Table S5. Survey questions and results - Interchangeability and switching considerations in tender design**		
Are biological reference products and biosimilars deemed interchangeable when formulating a tender?	Yes	51% (24/47)
	It depend on the product class	28% (13/47)
	No	13% (6/47)
	Other	9% (4/47)
	Mentioned open answer under "Other": depends on clinician agreement	
When tendering for biological medicines, are biosimilars grouped in the same lot as the reference product or in a different lot?	Grouped in the same lot	68% (32/47)
	In a different lot	13% (6/47)
	Other	19% (9/47)
	Mentioned open answers under "Other": depends on the product, grouped in same lot if safety of switch is documented, grouped in the same lot for new patients and in different lots for patients under treatment	
When tendering for biological medicines, is a difference made between bio-naïve patients and patients already under treatment with the biological medicine?	Yes	36% (17/47)
	No	43% (20/47)
	Other	21% (10/47)
	Mentioned open answers under "Other": depends on the switch documentation, depends on the product, depends on the clinicians	
When a patient already undergoes treatment with the previous winner…	There is an option to keep the patient on therapy with the previous winner	51% (24/47)
	The patient receives the tendered product instead of the previous winner	21% (10/47)
	Other	28% (13/47)
	Mentioned open answers under "Other": therapy with the previous winner is only continued on specific demand of patient and physician, it depends on the decision of the team, it depends on the case, the decision is taken during the consultation together with the patient, the patient can go back to the previous winner if side effects occur with the new product	

If the option to keep the patient on therapy with the previous winner exists, how is this product purchased?	Direct procurement of the previous winner	42% (10/24)
	Via a multiple winner tender, i.e. there are multiple winners and one of them is the previous winner	29% (7/24)
	Existing contract with the previous winner	21% (5/24)
	Other	8% (2/24)
	Mentioned open answer under "Other": open framework with all products listed	
