# Supplementary material for: Off-Patent Biologicals and Biosimilars Tendering in Europe—A Proposal towards More Sustainable Practices
Source: Pharmaceuticals (Basel). 2021 May 24;14(6):499. doi: 10.3390/ph14060499 (PMC8225063; doi:10.3390/ph14060499)
Supplement: Supplementary file 1 [file pharmaceuticals-14-00499-s001.zip › Supp Table S6.pdf]

Off-patent biologicals and biosimilars tendering in Europe – a proposal towards more sustainable practices

Pharmaceuticals – Biosimilars special issue

Liese Barbier, Steven Simoens, Caroline Soontjens, Barbara Claus, Arnold G. Vulto, Isabelle Huys

Corresponding author: Liese Barbier, Email: liese.barbier@kuleuven.be

**Supp. Table S6. Interview participants' characteristics**	
**Characteristics**	**Participants (N total = 28)**
	N
**Country**	
Pan-European	4
Belgium	4
Denmark*	3
France	4
Italy	4
Norway*	3
The Netherlands	4
England	3
**Stakeholder group**	
Hospital pharmacist/purchaser	16
Pharmaceutical industry	12
** One interviewee shared insights about both Norway and Denmark, and was therefore counted as participant for both countries in the table. For the total number of participants, the participant was only counted once.*	
Participants with a pan-European perspective often also provided (home) country specific insights and/or examples in addition to their pan-European perspective.	
Abbreviations: *N: number*	
