# Supplementary material for: Off-Patent Biologicals and Biosimilars Tendering in Europe—A Proposal towards More Sustainable Practices
Source: Pharmaceuticals (Basel). 2021 May 24;14(6):499. doi: 10.3390/ph14060499 (PMC8225063; doi:10.3390/ph14060499)
Supplement: Supplementary file 1 [file pharmaceuticals-14-00499-s001.zip › Supp Table S7.pdf]

Off-patent biologicals and biosimilars tendering in Europe – a proposal towards more sustainable practices

Pharmaceuticals – Biosimilars special issue

Liese Barbier, Steven Simoens, Caroline Soontjens, Barbara Claus, Arnold G. Vulto, Isabelle Huys

Corresponding author: Liese Barbier, Email: liese.barbier@kuleuven.be

Supp. Table S7. Interview participants' characteristics

Characteristics	Stakeholder group	Country
IV 1	Hospital pharmacy	France
IV 2	Hospital pharmacy	Belgium
IV 3	Procurement body	Denmark
IV 4	Pharmaceutical company	Pan-European insights
IV 5	Procurement body	Norway
IV 6	Consultancy organization	Pan-European insights
IV 7	Specialist pharmacy	England
IV 8	Hospital procurement entity	Belgium
IV 9	Pharmaceutical industry trade organization	Italy
IV 10	Pharmaceutical company	England
IV 11	Pharmaceutical company	Denmark and Norway
IV 12	Pharmaceutical company	France
IV 13	Pharmaceutical company	France
IV 14	Pharmaceutical company	Norway
IV 15	Regional specialist pharmacy	England
IV 16	Consultancy organization	Denmark
IV 17	Pharmaceutical company	Belgium
IV 18	Hospital pharmacy	The Netherlands
IV 19	Hospital pharmacy	The Netherlands
IV 20	Pharmaceutical industry trade organization	Pan-European insights
IV 21	Pharmaceutical company	The Netherlands
IV 22	Pharmaceutical industry trade organization	Pan-European insights
IV 23	Pharmaceutical company	Belgium
IV 24	Hospital pharmacy	France
IV 25	Hospital pharmacy	Italy
IV 26	Hospital pharmacy	Italy
IV 27	Hospital procuring entity	The Netherlands
IV 28	Hospital pharmacy	Italy

Participants with a pan-European perspective often also provided (home) country specific insights and/or examples in addition to their pan-European perspective.

Abbreviations: IV: interview
