# Supplementary material for: Off-Patent Biologicals and Biosimilars Tendering in Europe—A Proposal towards More Sustainable Practices
Source: Pharmaceuticals (Basel). 2021 May 24;14(6):499. doi: 10.3390/ph14060499 (PMC8225063; doi:10.3390/ph14060499)
Supplement: Supplementary file 1 [file pharmaceuticals-14-00499-s001.zip › Supp Table S8.pdf]

Off-patent biologicals and biosimilars tendering in Europe – a proposal towards more sustainable practices

Pharmaceuticals – Biosimilars special issue

Liese Barbier, Steven Simoens, Caroline Soontjens, Barbara Claus, Arnold G. Vulto, Isabelle Huys

Corresponding author: Liese Barbier, Email: liese.barbier@kuleuven.be

**Supp. Table S8. Recommendations extracted from position/white papers on tender procedures (for off-patent biological medicines and biosimilars) in Europe**	
**Supplier perspective**	
2012 - European Biopharmaceutical Enterprises (EBE)[27]	
Number of winners	- Ensure sufficiently broad choice of products<br>- "One winner takes all" could result in forced switching
Award criteria	Apply a variety award criteria and not only price
Stakeholder involvement	Involve a scientific committee, including physicians, in the decision-making process
Therapeutic freedom	Respect and safeguard physician's autonomy of clinical treatment choice
Switching considerations	- Allow treatment continuation<br>- Any switching should only happen under guidance of a treating physician in relation to the concerned patient
2015 - EuropaBio[28]	
Number of winners	- Procurement process should foster diversity of suppliers and allow multiple treatment choices, not limiting physician/patient to one treatment choice<br>- Winner takes all tenders should be avoided
Award criteria	- Additional criteria besides price should be considered<br>- Ensure transparency on definition of eligibility criteria and award decision<br>- Public procurement practices should ensuring that innovation is valued and appropriately rewarded
Stakeholder involvement	Purchasers should hold preliminary discussions with the different bidders
Therapeutic freedom	Allow freedom of choice for physicians and patients by ensuring that treatment options are not limited and decisions can always be made by the physician in consultation with the patient
Switching considerations	- It should be reflected in the public procurement design that biological medicines cannot be interchanged<br>- Respect physicians' prescribing authority to keep treated patients on current treatments<br>- Patients under treatment should not be switched
Supply considerations	- Consider complexity of manufacturing and allow for sufficient lead times

	- Apply volume commitments from purchasing authority<br>- Tender contract should be sufficiently stable and accurately predict demand over a sufficiently long period of time
INN or class level tenders	- The bundling of on- and off-patent biological medicines in a single tender should be avoided.<br>- Only include therapeutically equivalent products in any grouping of medicines in a potential tender lot
General	Tenders should be designed in such a way to guarantee fair competition between all suppliers. Transparency about award criteria and the decision-making process should be ensured
2018 - Medicines for Europe[19]	
Number of winners	Adjust number of winners to market, product and country characteristics<br>- Multi-winner tenders should be preferred<br>- Market could be divided into lots for the different winners (first winner gets 50%, second winner 30%,…)
Award criteria	Apply award criteria that consider other factors than price and ensure fair competition<br>- Ensure fair design and application of criteria<br>- Consider product-specific characteristic (*e.g.* in case of value added medicines such as pre-filled syringes)
Stakeholder involvement	Stimulate dialogue between procurement specialists and industry to better understand each other's needs and requirements
Supply considerations	- Prevent disproportionate penalties, the value of the penalty should be proportionate to the contract value<br>- Adjust penalties to the cause of the inability to supply and the product characteristics<br>- If the supply inability is due to external reasons, an exit clause or framework that allows manufacturers to find a solution should be applied<br>- Accurate provision of volumes to be supplied (min. and max. volume caps)<br>- No penalty in case of inability to supply unexpected volumes (*e.g.* not specified in procurement contract)<br>- Use extended lead times to ensure predictable supply<br>- Spread tender procedures throughout the year to accommodate manufacturing capacity
Opening of procedures	Procurement processes should open when originator medicines are about to lose their patent protection/loss of exclusivity, to ensure immediate competition and access for biosimilar competitors
General	- Procurement process and administrative procedures should be predictable, harmonised and transparent<br>- A holistic view should be taken when designing procurement processes
2017 - EuropaBio[29] (revision of 2015 position)	
Award criteria	- Apply MEAT award criteria<br>- Ensure transparency on definition of eligibility criteria and decision<br>- Contracting authorities should specify and publish award criteria and the relative weighting given to them in advance and on time so bidders can consider them when preparing their tenders
Stakeholder involvement	Apply a multi-disciplinary approach:<br>- Purchasing authorities should be supported by clinical experts, health economists and patients<br>- Expertise should be brought together in a tendering committee
INN or class level tenders	Ensure homogeneity of tendering lots, avoiding bundling of on- and off-patent biological medicines in a single tender

General	- Tender procedures should be designed in such a way that guarantees fair competition between all suppliers. Discrimination against originator manufacturers should be avoided.<br>- Tender procedures and the decision-making process should be widely advertised to ensure transparency and avoid risk of favouritism
2018 - IQVIA (formerly Quintiles and IMS Health) (report commissioned by Pfizer)[3]	
Number of winners	Apply multi-winner tenders, allowing greater prescription and product choice for physicians and patients, while sustaining healthier competition. Ensure that more than one competitor continues to supply the market. Single-winner tenders exert maximum price pressure but jeopardise sustainability
Award criteria	Include additional criteria beyond price, incentivizing manufacturers to innovate in areas to support patients and providers
Supply considerations	Multi-winner tenders can encourage multiple manufacturers to function within a market, reducing the potential risk of shortages
Opening procedures	Apply a short periodicity for tenders (12 months max)
2019 - Pugatch Consilium (report commissioned by European Federation of Pharmaceutical Industries and Associations (EFPIA))[66]	
Number of winners	Include the possibility of a variety of products from multiple suppliers (no ‘winner takes all’ tender)
Therapeutic freedom	Include option for physicians to opt-out individual patients based on medical needs at the physician’s discretion
Supply considerations	Ensure an effective supply term that ranges between min 12 and max 24 months
INN or class level tenders	Perform tenders solely at molecule level
2019 - KPMG (commissioned by Medicines for Europe)[35]	
Number of winners	Apply multi-winner tender models
Award criteria	Apply MEAT criteria
Stakeholder involvement	- Create guidelines and/or info campaigns to increase awareness of patients and HCP on biosimilars including on switching<br>- Implement benefit sharing methods
Switching considerations	Draft national/hospital guidelines on switching
Supply considerations	Set accurate estimates of volumes to be supplied
Opening of procedures	Swiftly reopen tenders after the entry of the first multisource medicine
General	Establish leaner tender procedures
**Purchaser perspective**	
2018 - European Association of Hospital Pharmacists (EAHP)[20]	
Award criteria	Integrate qualitative criteria, such as MEAT principles. Price driven negotiations could lead to medicine shortages and long-term rise of prices in “winner takes all” scenario. Consider all aspects, including *e.g.* cost/benefit, patient safety (*i.e.* through reduced look/sound-alike), company reliability factors (*e.g.* transport and distribution procedures).
Stakeholder involvement	Hospital pharmacists should lead tender procedures. Also at national/ regional level, hospital pharmacist’s view should be considered in negotiations by government
Supply considerations	Impact assessment tools and continuous monitoring of tendering should include supply chain vulnerability and sustainability considerations
General	Procurement process should be evaluated regularly and actions should be taken to improve outcomes at therapeutic, patient safety and efficiency level

<u>Abbreviations:</u> HCP: healthcare professional, INN: international non-proprietary name, MEAT: Most Economically Advantageous Tender

The Public procurement of medicinal products White paper of Bird & Bird (2014), providing an overview of similarities and divergences in the applicability of public procurement legislation in the healthcare sector, across jurisdictions in the EU, is not included in this tabular overview as it does not include recommendations. [18]
