# Supplementary material for: Off-Patent Biologicals and Biosimilars Tendering in Europe—A Proposal towards More Sustainable Practices
Source: Pharmaceuticals (Basel). 2021 May 24;14(6):499. doi: 10.3390/ph14060499 (PMC8225063; doi:10.3390/ph14060499)
Supplement: Supplementary file 1 [file pharmaceuticals-14-00499-s001.zip › Supp Table S9.pdf]

Off-patent biologicals and biosimilars tendering in Europe – a proposal towards more sustainable practices

Pharmaceuticals – Biosimilars special issue

Liese Barbier, Steven Simoens, Caroline Soontjens, Barbara Claus, Arnold G. Vulto, Isabelle Huys

Corresponding author: Liese Barbier, Email: liese.barbier@kuleuven.be

**Supp. Table S9. Main topics addressed during the qualitative semi-structured expert interviews**
1. Views about the current organization of tenders for off-patent biologicals and biosimilars: opportunities, challenges, learnings, best-practices, examples
2. Views and recommendations about how to improve tender processes for off-patent biologicals and biosimilars
3. Views about the sustainability of current tender practices and the broader context of the off-patent biologicals market
4. Questions related to the design of tender procedures: the use of selection-and award criteria, national *versus* regional *versus* local tender organizational level, transparency of the process, guidance and information needs, the role of the physician and freedom of prescription, shortages, interchangeability and switching, future outlook
